# Homeostatic pruning and activity of epidermal nerves are dysregulated in barrier-impaired skin during chronic itch development

**DOI:** 10.1038/s41598-019-44866-0

**Published:** 2019-06-13

**Authors:** Sonoko Takahashi, Azusa Ishida, Akiharu Kubo, Hiroshi Kawasaki, Sotaro Ochiai, Manabu Nakayama, Haruhiko Koseki, Masayuki Amagai, Takaharu Okada

**Affiliations:** 1 Laboratory for Tissue Dynamics, RIKEN Center for Integrative Medical Sciences, Yokohama, Kanagawa 230-0045 Japan; 2 Graduate School of Medical Life Science, Yokohama City University, Yokohama, Kanagawa 230-0045 Japan; 3 Department of Dermatology, Keio University School of Medicine, Shinjuku-ku, Tokyo, 160-8582 Japan; 4 Laboratory for Skin Homeostasis, RIKEN Center for Integrative Medical Sciences, Yokohama, Kanagawa 230-0045 Japan; 5 Disease Biology Group, RIKEN Medical Sciences Innovation Hub Program, Yokohama, Kanagawa 230-0045 Japan; 6 Department of Frontier Research and Development, Kazusa DNA Research Institute, Kisarazu, Chiba 292-0818 Japan; 7 Laboratory for Developmental Genetics, RIKEN Center for Integrative Medical Sciences, Yokohama, Kanagawa 230-0045 Japan; 8 JST, PRESTO, Kawaguchi, Saitama 332-0012 Japan

**Keywords:** Fluorescence imaging, Fluorescence imaging, Somatic system, Somatic system, Somatic system

## Abstract

The epidermal barrier is thought to protect sensory nerves from overexposure to environmental stimuli, and barrier impairment leads to pathological conditions associated with itch, such as atopic dermatitis (AD). However, it is not known how the epidermal barrier continuously protects nerves for the sensory homeostasis during turnover of the epidermis. Here we show that epidermal nerves are contained underneath keratinocyte tight junctions (TJs) in normal human and mouse skin, but not in human AD samples or mouse models of chronic itch caused by epidermal barrier impairment. By intravital imaging of the mouse skin, we found that epidermal nerve endings were frequently extended and retracted, and occasionally underwent local pruning. Importantly, the epidermal nerve pruning took place rapidly at intersections with newly forming TJs in the normal skin, whereas this process was disturbed during chronic itch development. Furthermore, aberrant Ca^2+^ increases in epidermal nerves were induced in association with the disturbed pruning. Finally, TRPA1 inhibition suppressed aberrant Ca^2+^ increases in epidermal nerves and itch. These results suggest that epidermal nerve endings are pruned through interactions with keratinocytes to stay below the TJ barrier, and that disruption of this mechanism may lead to aberrant activation of epidermal nerves and pathological itch.

## Introduction

The skin barrier protects from invasions of external agents, and regulate immune and, most likely, nerve responses to them^1–3^. There are two important barrier structures in the epidermis of the skin, the cornified layer and tight junctions (TJs) formed in the granular layer (stratum granulosum, SG) right underneath the cornified layer. The genetic polymorphism affecting the cornified layer integrity has been found to be a causal factor of atopic dermatitis (AD)^1^. In addition, decreased expression of the key TJ protein has been suggested to contribute to the development of AD^4–6^.

Epidermal nerve fibers are unencapsulated nerve endings that detect noxious stimuli to conduct pain. In addition, sensory neurons innervating the epidermis are involved in the itch induction^7,8^. Although unproven, it has been suspected that epidermal nerves are aberrantly activated by overexposure to environmental stimuli during the development of AD due to the impaired protection by the epidermal barrier^2,3^. For the protection of epidermal nerves, the barrier structure should homeostatically cover over the nerves. However, anatomical relationship between the barrier structures and epidermal nerves during turnover of the epidermis has not been demonstrated.

In the present study, we show that epidermal sensory nerves are contained underneath keratinocyte TJs in the normal skin, which appears to be at least partly due to nerve pruning at newly forming TJs. Our data also suggest that during the development of chronic itch caused by epidermal barrier impairment, this dynamic anatomical relationship is disrupted, and epidermal nerves are aberrantly activated.

## Results

### Anatomical relationship between epidermal nerve endings and keratinocyte TJs in the normal and AD conditions

**Figure 1:**
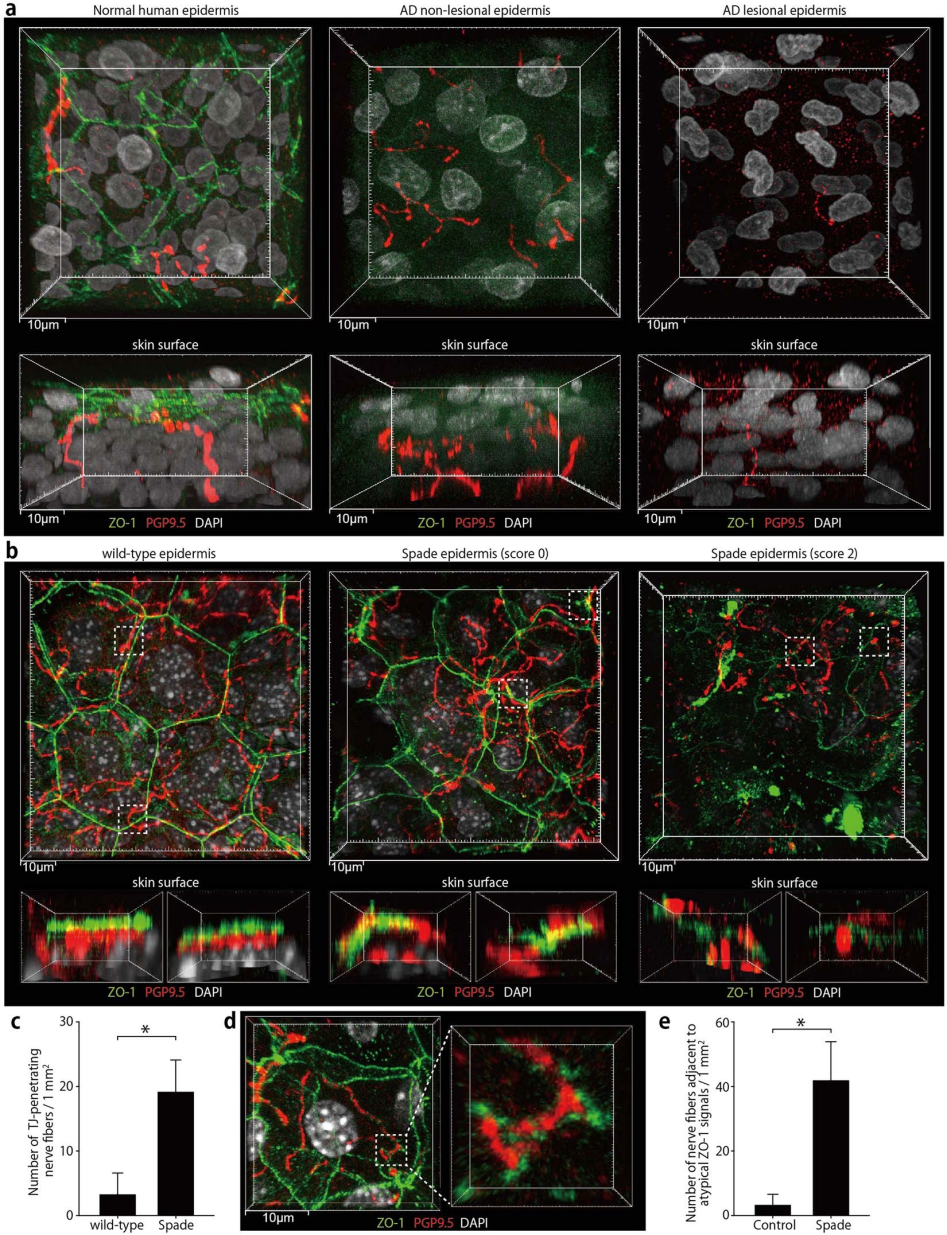
Epidermal nerve endings are contained under TJs in the normal human and mouse skin but not in the human AD or *Spade* mouse skin. (**a**) Whole-mount confocal fluorescence images of the healthy human epidermis and the epidermis of AD patients. PGP9.5^+^ nerve fibers and TJs visualized as ZO-1 localization are shown in vertical (upper, 44 μm projection depth) and horizontal (lower, 61.5 μm projection depth) projection images. See also Supplementary Movie 1. (**b**) Whole-mount confocal fluorescence images of the ear epidermis of wild-type and *Spade* mice without (score 0) or with lesions (score 2). The upper images are the vertical projection (12.5 and 21–22 μm projection depth for the wild-type and *Spade* mice, respectively). The lower images show horizontal views of the dashed square regions from the right side in the vertical projection images. See also Supplementary Movie 2. (**c**) The number of nerve fibers penetrating TJs, normalized by the epidermis area. (**d**) Whole-mount confocal fluorescence images of the SG of the Spade epidermis showing atypical ZO-1 accumulations around a nerve fiber. (**e**) the area-normalized number of nerve fibers surrounded by atypical ZO-1 accumulations. The data are shown as the mean ± s.e.m. in **c** and **e** (WT: n = 9, Spade: n = 20). *p < 0.05.

We first investigated the microanatomical relationship of epidermal nerves with the barrier structure in the normal human skin. Because epidermal nerves were reported to be extended up to the SG in the mouse skin^7,9^, we visualized TJs together with nerves by the whole mount immunofluorescence staining for the TJ protein ZO-1 and pan-neuronal marker PGP9.5. Indeed, we found nerve fibers were present in such close proximity to TJs that some of them might be in contact with each other. Interestingly, however, none of the nerve fibers were found to penetrate the TJs in the normal human skin (Fig. 1a; Supplementary Fig. 1a; Supplementary Movie 1). In the lesional skin of AD patients (see Methods for clinical characteristics of the lesions), ZO-1 localization at TJs was undetectable as previously reported (Fig. 1a; Supplementary Fig. 1a; Supplementary Movie 1)^10^. In severely lesioned skin areas, which had highly autofluorescent sediments, possibly of exudates over the epidermis, nerve fibers reaching the upper region of the thickened epidermis were scarcely found as described in the previous study (Supplementary Fig. 1a)^11^. In other lesional areas, nerve fibers were found in the upper epidermal region that did not show clear ZO-1 localization at TJs (Fig. 1a; Supplementary Movie 1). Even in the non-lesional skin of AD patients, there were areas where ZO-1 localization at TJs was severely impaired, which is consistent with the previous reports^4,10^, and nerves were found in the upper epidermal region (Fig. 1a; Supplementary Fig. 1a; Supplementary Movie 1).

In the normal mouse skin, as in the normal human skin, we found that epidermal nerves were in close proximity to TJs and kept below TJs. TJ-penetrating nerve fibers were scarcely found in the normal epidermis (Fig. 1b,c; Supplementary Movie 2). Penetration of TJs by processes of activated Langerhans cells were observed after tape stripping of the cornified layer as previously reported^12^. Even in this condition, nerves were kept below TJs (Supplementary Fig. 1b). Then we examined the positioning of nerve endings in an AD model, *Spade* (*Jak1*^*Spade/Spade*^) mice, in which the point mutation in Jak1 causes epidermal barrier impairment^13^. *Spade* mice started to develop spontaneous dermatitis of the ear skin in the specific pathogen free condition between 7 and 16 weeks after birth as previously reported^13^ (Supplementary Fig. 1c,d). By using Elizabethan collars, we found that the development of *Spade* dermatitis lesions was dependent on scratching (Supplementary Fig. 1d). In the lesioned area of the dermatitis skin (score 1 or higher), the epidermis was often destroyed, and the dermal nerve structure was disrupted, presumably by scratching (Supplementary Fig. 1e). The disruption of dermal nerves was not observed in the unscratched *Spade* ear without lesions (score 0). However, in the epidermis of 7-week-old or older *Spade* mice that were yet to show the abnormal scratching behavior (score 0), we found areas where SG keratinocytes had irregular shapes and their ZO-1 localization at TJs appeared less organized (Fig. 1b; Supplementary Fig. 1f; Supplementary Movie 2). At this pre-disease stage of the *Spade* epidermis, nerves were occasionally observed to penetrate TJs (Fig. 1b,c; Supplementary Fig. 1f). Additionally, in these mice, atypical accumulations of ZO-1 signals that did not appear to be a part of TJs were found around epidermal nerve fibers (Fig. 1d,e). In the lesional skin with progressed dermatitis, the areas where the epidermis was not yet demolished by scratching showed an impaired ZO-1 localization at TJs, resembling human AD skin (Fig. 1b; Supplementary Movie 2). Taken together, the above observations in human and mouse skin suggest that epidermal nerves may not be protected under the TJ barrier during and after the development of AD.

### Involvement of epidermis-innervating neurons in itch of *Spade* mice

**Figure 2:**
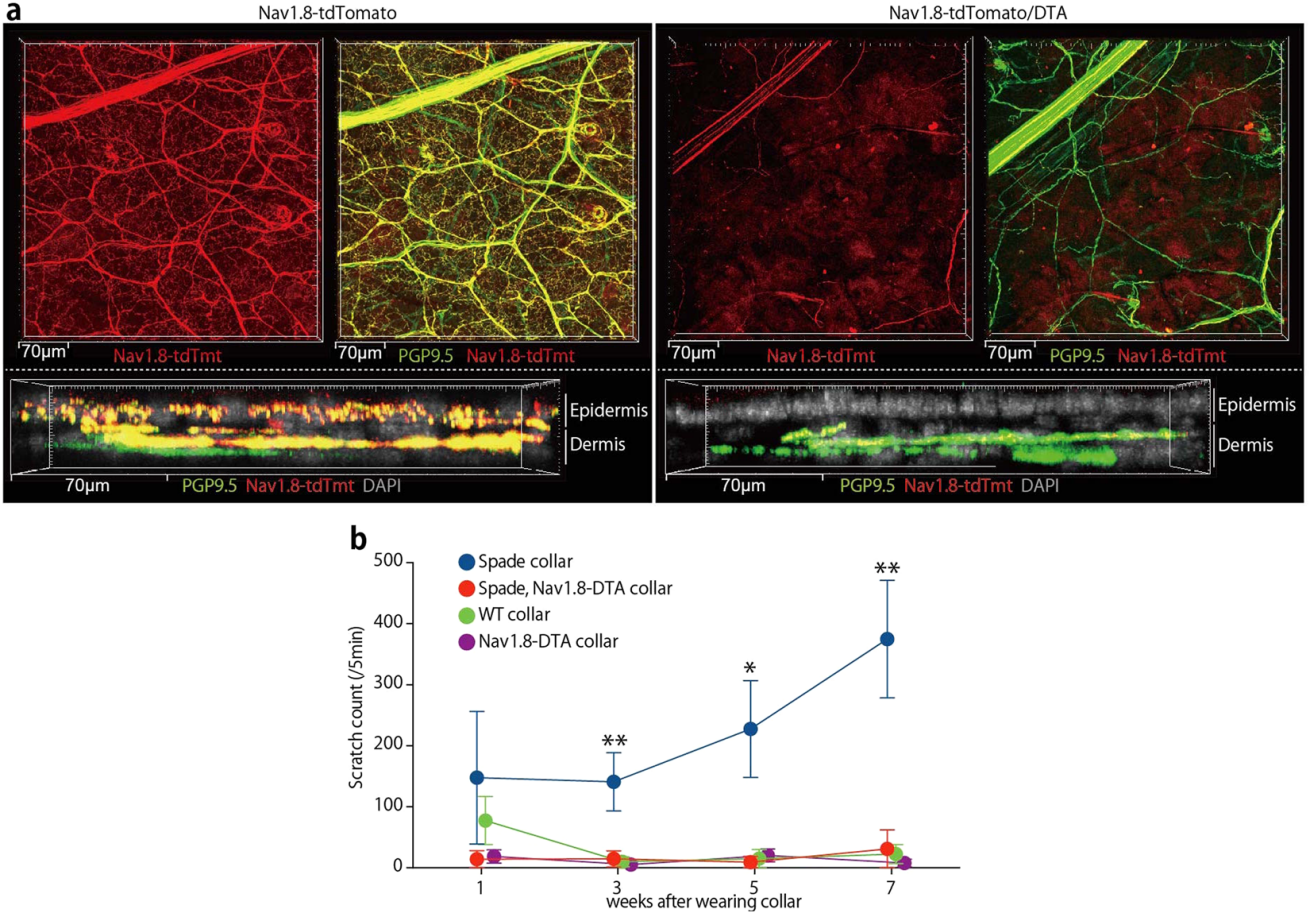
Epidermal nerve fibers are of Nav1.8-tdTomato^+^ sensory neurons, which are necessary for itch in *Spade* mice. (**a**) Whole-mount confocal fluorescence images of the ear skin from Nav1.8-tdTomato and Nav1.8-tdTomato/DTA mice. Nav1.8-tdTomato^+^ PGP9.5^+^ nerves and Nav1.8-tdTomato^−^ PGP9.5^+^ nerves are shown in vertical (top, 33 μm projection depth) and horizontal (bottom, 28.4 μm projection depth) projection images. See also Supplementary Fig. 2. (**b**) The number of scratching strokes by the hind paws of the indicated mice after wearing Elizabethan collars. The data are shown as the mean ± s.e.m. (n = 3–4). *p < 0.05, **p < 0.01.

In order to further characterize epidermal nerves, we analyzed Nav1.8-Cre Rosa26-CAG-flox-stop-tdTomato (Nav1.8-tdTomato) mice^14,15^ because epidermal nerves are thought to be mainly nociceptors, which express Nav1.8 sodium channels^16,17^. Indeed, in the whole mount ear skin, most, if not all, of the epidermal nerve fibers stained for PGP9.5 appeared to contain Nav1.8-tdTomato, while in the dermis both Nav1.8-tdTomato-positive nerves and -negative nerves were found (Fig. 2a; Supplementary Fig. 2). This indicates that virtually all neurons that innervate the epidermis express or have expressed Nav1.8. The notion was confirmed by further crossing the mice with Rosa26-flox-stop-diphteria toxin A (DTA) mice^18^. In the resultant mice, epidermal nerves were completely depleted whereas Nav1.8-tdTomato-negative nerves remained in the dermis. In addition, a small number of Nav1.8-tdTomato-positive nerves remained in the dermis (Fig. 2a; Supplementary Fig. 2). These nerves might have expressed Nav1.8-Cre weakly and/or transiently, which might have been sufficient for tdTomato expression from the CAG-inserted Rosa26 knock-in allele, with a possibly increased chromatin accessibility, but not for DTA expression from the other Rosa26 knock-in allele without CAG.

To examine the involvement of Nav1.8^+^ neurons in the development of *Spade* itch, we crossed *Spade* mice to Nav1.8-DTA mice. Regardless of the *Spade* mutation, Nav1.8-DTA mice were insensitive to pain, and after reaching adulthood, frequently injured themselves on various parts of the skin most likely during grooming, which complicated the analysis of the skin lesion severity. Therefore, we put collars on them after 6 weeks of birth to prevent self-injury, and then analyzed their scratching behavior. Strikingly, Nav1.8-DTA *Spade* mice did not show intense scratching behavior that conventional *Spade* mice showed (Fig. 2b). Therefore, Nav1.8^+^ neurons including epidermis-innervating neurons contain a population necessary for the itch development of *Spade* mice.

### Dynamic nature of epidermal nerve endings

**Figure 3:**
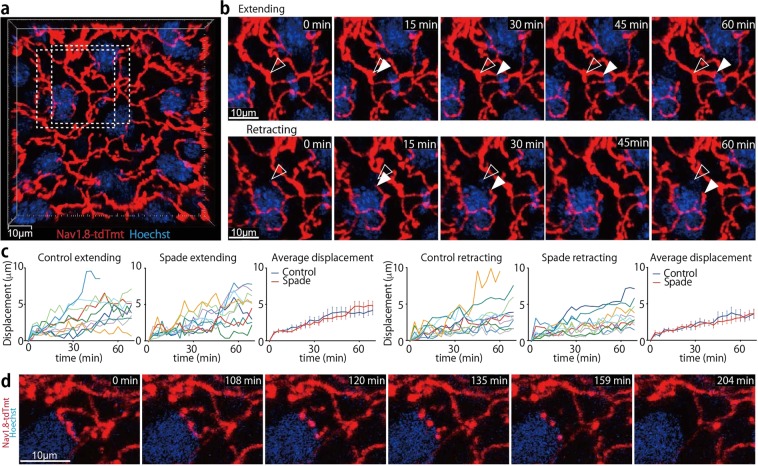
Epidermal nerve endings are frequently extended, retracted, and occasionally pruned. (**a**) An intravital multiphoton image (16 μm projection depth) of nerves in the SG of an Nav1.8-tdTomato mouse. (**b**) Extending and retracting nerve endings in the dashed rectangles in A. Open and filled arrowheads indicate the positions of nerve endings at 0 min and each later time point, respectively. See also Supplementary Movie 3. (**c**) Displacement of extending and retracting nerve endings. In the “Average displacement” graphs, data are shown as the mean ± s.e.m. (n = 10). (**d**) Time-lapse images (14 μm projection depth) of epidermal nerve pruning. See also Supplementary Movie 4.

Nav1.8-tdTomato-positive nerve fibers in the epidermis were readily detectable by intravital multiphoton or confocal microscopy (Fig. 3a; Supplementary Fig. 3a,b). The length of nerve fibers contained per unit volume of the SG was reduced in Spade mice over 7 weeks old that had not developed scratch lesions (score 0), compared to control mice (Supplementary Fig. 3c). However, this appeared to be mainly because of a thickened epidermis of the Spade mice. In the Spade epidermis, SG keratinocytes were morphologically less flattened than normal SG keratinocytes. As a result, the SG of the Spade mice was approximately 2.4-fold thicker than that of control. Therefore, the total length of nerve fibers in the SG per unit area of the skin was not significantly different between the Spade mice and control mice (Supplementary Fig. 3c).

By time-lapse imaging analysis, we found that nerve endings in the normal epidermis are dynamic, constantly extending and retracting up to 10 μm in an hour (Fig. 3b,c; Supplementary Movie 3). Extension and retraction speeds of nerve endings were not significantly different between control and Spade mice (Fig. 3c). In addition, we observed that epidermal nerves of control mice occasionally underwent local fragmentation into pieces, and these pieces subsequently dissipated (Fig. 3d; Supplementary Movie 4). The fragmentation followed by dissolution of local nerve fibers, hereafter called ‘pruning’, spanned the mean fiber length of 53 μm (the standard deviation 32 μm, n = 10), and was found only for epidermal nerves, but not for the dermal nerves. Pruning of epidermal nerves was observed in *Spade* mice as well, though its spatiotemporal regulation appeared abnormal in *Spade* mice older than 7 weeks as described below. These results suggest that epidermal nerve endings are constitutively remodeled by their extension, retraction, and pruning.

### Epidermal nerve pruning at newly formed TJs

**Figure 4:**
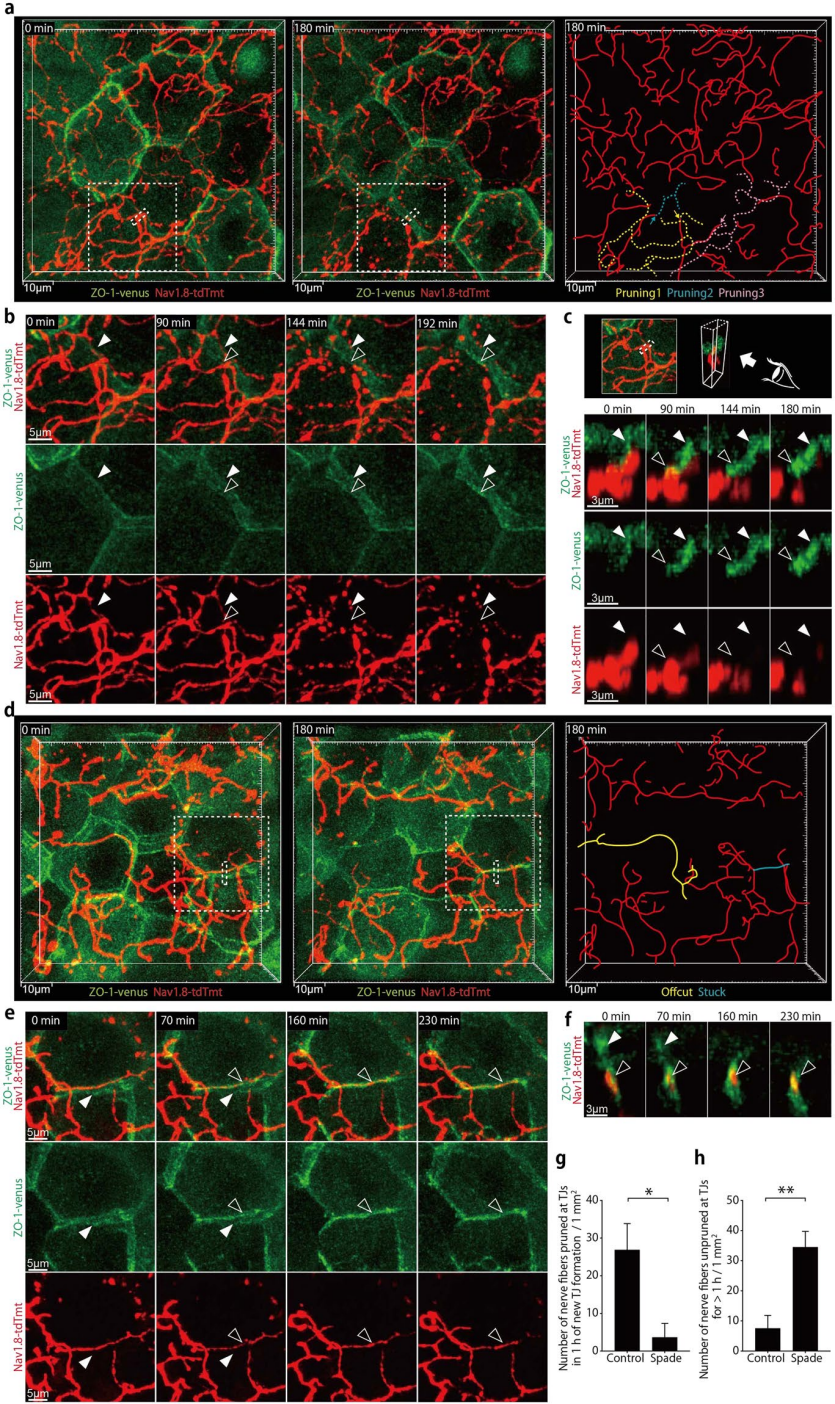
Epidermal nerve endings are rapidly pruned at newly formed TJs in the normal skin but not in the *Spade* skin around the disease onset. (**a**) *In vivo* time-lapse confocal images (9.5 μm projection depth) of epidermal nerves and TJs in an Nav1.8-tdTomato ZO-1-Venus mouse (far-left and middle panels). The far-right drawing indicates the locations of nerve branches that underwent fragmentation (yellow, blue, and pink dashed lines). The yellow and blue ones were pruned at the newly forming TJ. The pink one was pruned at a non-TJ site (see Supplementary Fig. 4). The sites of nerve pruning are indicated by the arrows. See also Supplementary Movie 5. (**b**) Time-lapse images of the new TJ formation and nerve pruning in the region demarcated by the dashed squares in (**a**) See also Supplementary Movie 6. (**c**) Time-lapse, horizontal views of the region demarcated by the dashed rectangles in **a** (3.1 μm projection depth). (**d**) An intravital confocal image of the ear epidermis from a *Spade* Nav1.8-tdTomato ZO-1-Venus mouse (20 μm projection depth). See also Supplementary Movie 7. (**e**) Time-lapse images of the region demarcated by the dashed square in (**d**) (11 μm projection depth). See also Supplementary Movie 8. (**f**) Time-lapse, horizontal views of the region demarcated by the dashed rectangle in (**d**) (3.7 μm projection depth). Open and filled arrowheads indicate the new and old TJs, respectively. (**g**) The area-normalized number of nerve fibers pruned at new TJs in an hour of the new TJ formation. (**h**) The area-normalized number of nerve fibers unpruned at TJs without being pruned for more than an hour. The data are shown as the mean ± s.e.m. (WT: n = 4, *Spade*: n = 3). *p < 0.05, **p < 0.01.

It seemed possible that the constitutive remodeling of epidermal nerve endings might contribute to the maintenance of their anatomical relationship with the TJ barrier during the turnover of the epidermis. To visually address this possibility, we crossed Nav1.8-tdTomato mice with ZO-1-Venus transgenic mice whose epidermal TJs are labeled with recombinant ZO-1 fused to the Venus fluorescenct protein (ZO-1-Venus), and conducted intravital confocal imaging of the epidermis. As reported previously, sporadic appearance and disappearance of TJ polygons were observed (Fig. 4a; Supplementary Movie 5)^19^. New TJs were formed alongside old TJs, in slightly lower Z-axis positions than old ones (Fig. 4b,c; Supplementary Movie 6). Interestingly, when new TJs were formed at positions where nerve fibers lay, nerve fibers appeared to be severed at the intersections with new TJs typically within an hour, and nerve fiber offcuts immediately became fragmented into pieces (Fig. 4b,c; Supplementary Movie 6). These results suggest that epidermal nerves were pruned at newly forming TJs to be constantly contained under the barrier structure.

We then analyzed ZO-1-Venus Nav1.8-tdTomato mice that carried the homozygous *Spade* mutation. In line with the ZO-1 immunofluorescence images (Fig. 1b; Supplementary Fig. 1f), these mice after 7 weeks of birth had microscopically abnormal skin areas, in which the polygonal shapes of keratinocytes outlined by ZO-1-Venus signals appeared to be distorted (Fig. 4d; Supplementary Movie 7). In these skin areas, some nerves were found to penetrate newly formed TJs, outlined by ZO-1-Venus localization, without becoming fragmented for more than an hour, sometimes several hours (Fig. 4e–h; Supplementary Movie 8). We also observed nerve fiber offcuts that did not immediately break into pieces and remained for more than 20 min (Supplementary Fig. 4a; Supplementary Movie 9). These results suggest that in *Spade* mice nearing the disease onset, epidermal nerve pruning at newly formed TJs does not take place as rapidly after the new TJ formation as in the normal skin.

We also noticed that nerve pruning occasionally took place near TJs but not exactly at positions where new TJs were formed in the normal epidermis. Interestingly, in this case, punctate ZO-1-Venus accumulations were transiently formed at the position and timing of the nerve pruning (Supplementary Fig. 4b,c; Supplementary Movie 10). The transient and punctate ZO-1-Venus accumulations were also observed on nerve fibers of the *Spade* epidermis. However, nerve fibers were not pruned at the transient ZO-1-Venus accumulations in the *Spade* epidermis (Supplementary Fig. 4d,e; Supplementary Movie 11). These results suggest that keratinocytes may also interact with nerves at non-TJ sites to remodel the layout of epidermal nerve fibers, and that this process is also disturbed in the *Spade* skin.

### Aberrant Ca^2+^ increases in sensory nerves in barrier-impaired skin during itch development

**Figure 5:**
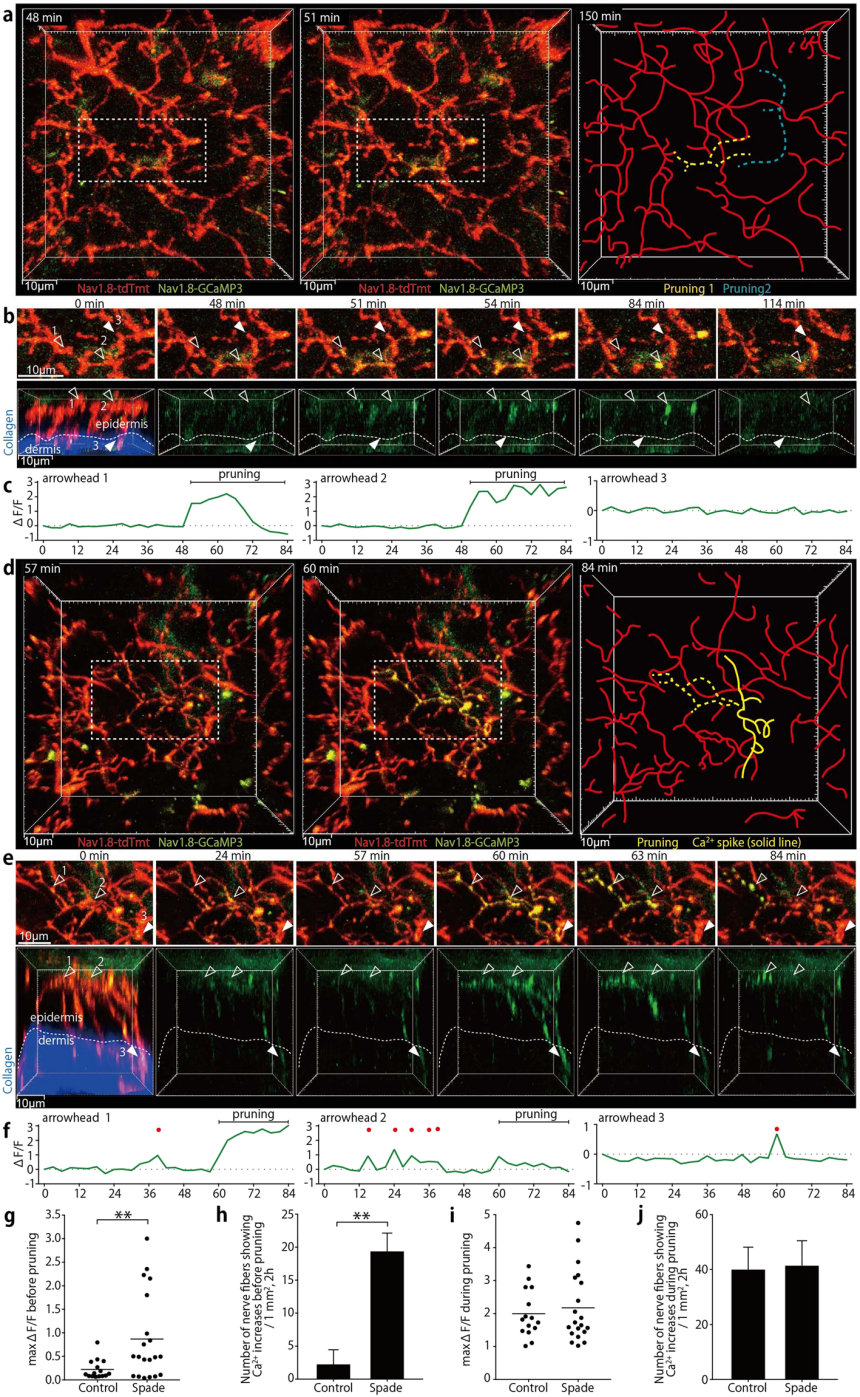
Aberrant nerve Ca^2+^ increases are observed in association with epidermal nerve pruning in *Spade* mice around the disease onset. (**a**) *In vivo* time-lapse multiphoton images (19 μm projection depth) of epidermal and dermal nerves in an Nav1.8-tdTomato/GCaMP3 mouse (far-left and middle). The dashed rectangles indicate the region shown in. (**b**) The far-right drawing indicates the locations of nerve branches that underwent fragmentation (yellow and blue dashed lines). (**b**) Local Ca^2+^ increases in epidermal nerve branches that underwent pruning (open arrowheads). Filled arrowheads indicate one of dermal fibers in which Ca^2+^ increases were not observed. The upper sequence shows vertical projection views. In the lower sequence of horizontal views, tdTomato and dermal second harmonic signals are shown only in the 0-min time-point image. See also Supplementary Movie 12. **(c)** Relative fluorescence intensity changes (∆F/F) in the GCaMP3 channel in 2^3^ μm3 regions indicated by the arrowheads in (**b**). (**d**) *In vivo* time-lapse multiphoton images (47 μm projection depth) of epidermal and dermal nerves in a *Spade* Nav1.8-tdTomato/GCaMP3 mouse (far-left and middle). The dashed rectangles indicate the region shown in (**e**) The yellow dashed line in the far-right drawing indicates an epidermal nerve branch that showed repetitive Ca^2+^ increases and underwent fragmentation. The yellow solid line indicates a nerve part that showed a Ca^2+^ spike without undergoing fragmentation. (**e**) Repetitive Ca^2+^ increases in an epidermal nerve branch that underwent pruning (open arrowheads) and a Ca^2+^ spike that reached a dermal nerve fiber (filled arrowheads). The upper sequence shows vertical projection views. In the lower sequence of horizontal views, tdTomato and dermal second harmonic signals are shown only in the 0-min time-point image. See also Supplementary Movie 13. (**f**) Relative fluorescence intensity changes (∆F/F) in the GCaMP3 channel in 2^3^ μm^3^ cubic regions indicated by the arrowheads in (**e**) Red dots indicate time points when ∆F/F was > 0.5. (**g**) Maximum fluorescence intensity changes in the GCaMP3 channel before pruning. (**h**) The area- and time-normalized number of nerve fibers showing Ca^2+^ increases (max ∆F/F >0.5) before pruning. (**i**) Maximum fluorescence intensity changes in the GCaMP3 channel during pruning. (**j**) The area- and time-normalized number of nerve fibers showing Ca^2+^ increases (max ∆F/F >0.5) during pruning. The data are shown as the mean ± s.e.m. in (**h**) and (**j**) (WT: n = 3, *Spade*: n = 3). *p < 0.01.

To examine nerve activities associated with the normal or defective pruning of epidermal nerves, we performed Ca^2+^ imaging analysis of skin nerves in mice that carried the Rosa26-CAG-flox-stop-GCaMP3 allele^20^ in addition to the Nav1.8-Cre and Rosa26-CAG-flox-stop-tdTomato alleles. Interestingly, the epidermal nerve pruning was always accompanied by local, transient Ca^2+^ increases. In the control mouse epidermis, the Ca^2+^ increases in nerve fibers undergoing pruning were observed only concurrently with the pruning, and were always confined in parts pruned away (Fig. 5a–c; Supplementary Movie 12). Longer-range Ca^2+^ spikes suggestive of spontaneous action potentials were never observed in the control skin.

In *Spade* mice over 7 weeks of age, however, we observed transient and, in three recordings, repetitive Ca^2+^ increases in epidermal nerves 6 to 90 min before the epidermal nerves underwent pruning (Fig. 5d–h; Supplementary Movie 13). Occasionally, longer-range Ca^2+^ spikes that reached dermal nerves were also observed (Fig. 5d–f; Supplementary Movie 13). The Ca^2+^ increases preceding epidermal nerve pruning was far more evident in the adult *Spade* mice than in age-matched control mice (Fig. 5g,h), whereas Ca^2+^ increases coinciding with pruning were similar in both types of mice (Fig. 5i,j). By contrast, in young *Spade* mice at 4–6 weeks old, Ca^2+^ increases associated with epidermal nerve pruning were not significantly different from those in young control mice (Supplementary Fig. 5). Thus, around the onset of pathological itch, aberrant Ca^2+^ increases are induced in epidermal nerves before their pruning in *Spade* mice.

**Figure 6:**
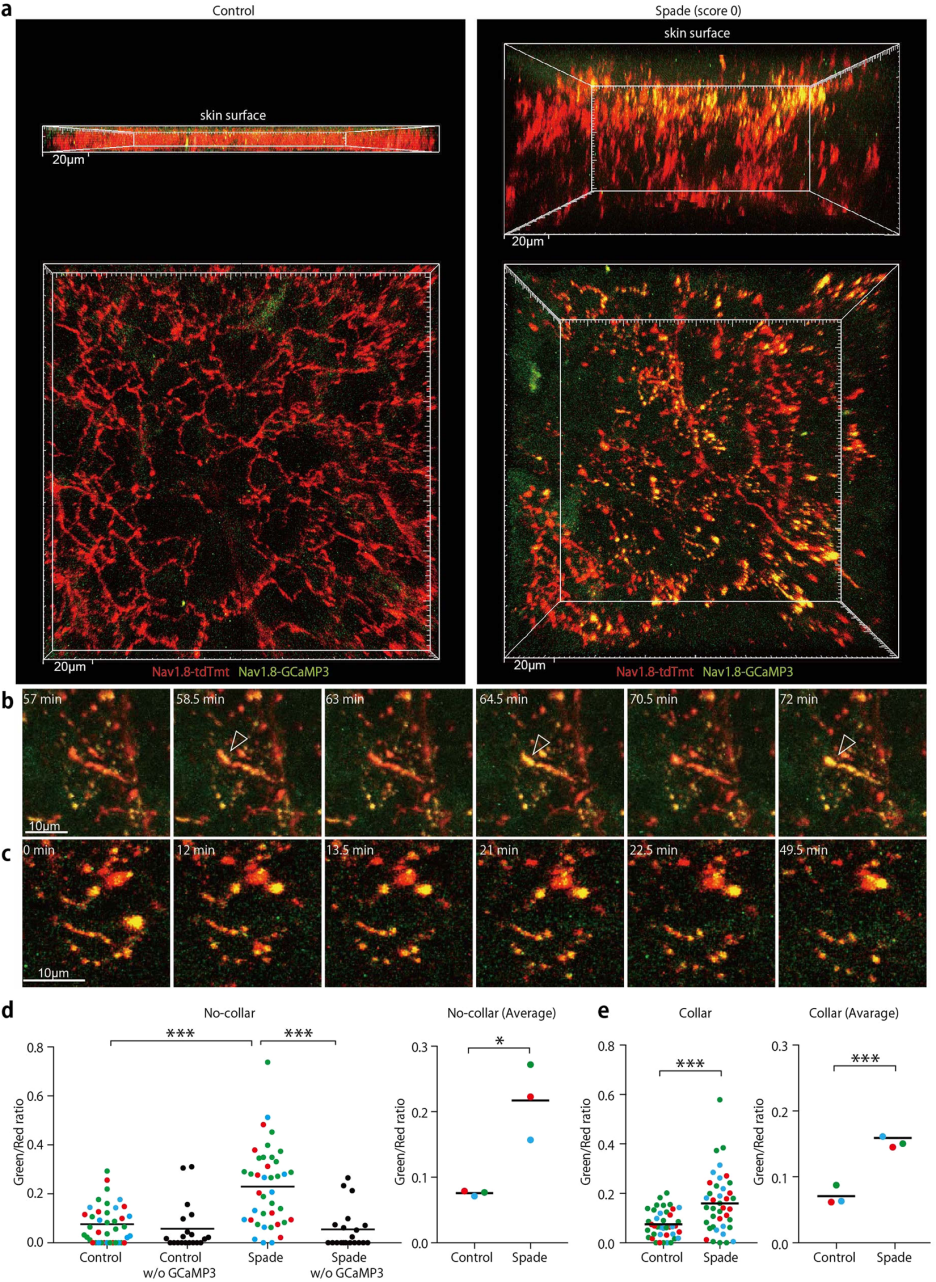
Sustained Ca^2+^ increases are induced in epidermal nerve fibers in the itching skin of *Spade* mice independently of scratching. (**a**) Intravital multiphoton images of the ear epidermis from control Nav1.8-tdTomato/GCaMP3 and *Spade* (score 0) Nav1.8-tdTomato/GCaMP3 mice. Upper and lower images are horizontal and vertical projection views, respectively. See also Supplementary Movie 14. (**b**,**c**) Time-lapse images of an epidermal nerve fiber showing repetitive Ca^2+^ increases (arrowheads in b) and fragmented nerve fibers with increased Ca^2+^ (**c**) in the *Spade* (score 0) mouse. See also Supplementary Movie 15. (**d**) Fluorescence intensity ratio of GCaMP3 to tdTomato. Shown are the data at the individual positions in the epidermal nerve fibers (left) and the averaged data from each experiment (right). Different colors of the data points indicate different mice in each group. Negative control data from Nav1.8-tdTomato and *Spade* (score 0) Nav1.8-tdTomato mice without the GCaMP3 allele are also shown in the left graph. (**e**) Fluorescence intensity ratio of GCaMP3 to tdTomato in epidermal nerves of mice that had worn Elizabethan collars. *p < 0.05, ***p < 0.001.

In some skin areas of *Spade* mice over 8 weeks, Ca^2+^ concentration in epidermal nerves especially in the upper epidermal region was remarkably elevated in a sustained manner from the beginning of imaging (Fig. 6a; Supplementary Movie 14). In some nerve endings, Ca^2+^ concentration was again oscillating in the range higher than control levels (Fig. 6b; Supplementary Movie 15). Extensive fragmentation of nerve fibers was also observed in these skin areas (Fig. 6c; Supplementary Movie 15). The sustained Ca^2+^ increases in epidermal nerves were significant in the *Spade* ear skin without visible lesions from scratching (Fig. 6d). Nevertheless, it was still possible that the Ca^2+^ increase might be a result of scratching. To test this possibility, we put Elizabethan collars on *Spade* mice and control mice from 6 weeks of birth, and waited for *Spade* mice to show intense scratching behavior after 7 weeks of birth. The imaging analysis of these mice revealed that Ca^2+^ concentration in epidermal nerves was increased in the itching, yet unscratched skin of collared *Spade* mice (Fig. 6e). Thus, it is suggested that in the skin with barrier impairment, epidermal nerve endings are stimulated near TJs before scratching further affects the epidermal barrier function and nerve activity.

In order to investigate if epidermal nerve Ca^2+^ increases generally accompany itch caused by skin barrier impairment, we used another dry skin model in which the sequential topical application of acetone/ether and water (AEW) induces barrier disruption and itch^21–23^. Repetitive AEW application on the ear skin of collared Nav1.8-tdTomato/GCaMP3 mice for more than a week induced dry skin and itch (Supplementary Fig. 6a), which appeared moderate compared to *Spade* itch (Supplementary Fig. 2b). Interestingly, in the itchy yet unscratched ear epidermis, ZO-1 expression at TJs was attenuated (Supplementary Fig. 6b), and Ca^2+^ in epidermal nerves was mildly but significantly increased, again in the upper epidermal region (Supplementary Fig. 6c,d). These data further suggest the spatial linkage between the Ca^2+^ increase in epidermal nerves and TJ barrier impairment during the development of chronic itch.

**Figure 7:**
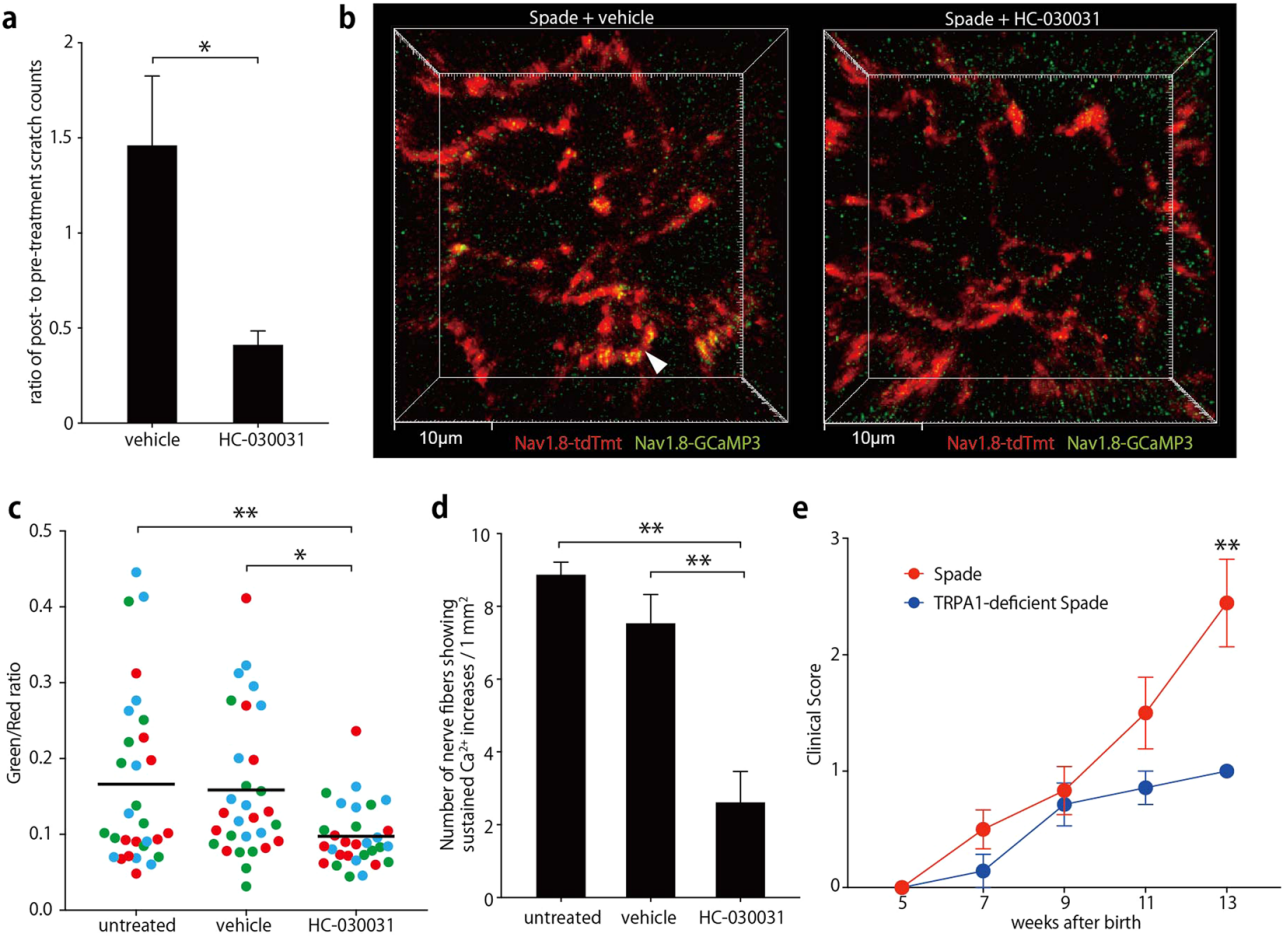
Inhibition of TRPA1 attenuates epidermal nerve Ca^2+^ increases and itch in *Spade* mice. (**a**) Ratio of ear scratch counts after to before topical treatment of the Spade ear skin with vehicle or HC-030031. (**b**) Intravital multiphoton images (16 μm projection depth) of the ear epidermis of *Spade* Nav1.8-tdTomato/GCaMP3 mice after treatment with vehicle or HC-030031. (**c**) Fluorescence intensity ratio of GCaMP3 to tdTomato. Shown are the data at the individual positions in the epidermal nerve fibers. Different colors of the data points indicate different mice in each group. (**d**) The area-normalized number of nerve fibers showing Ca^2+^ increases (fluorescence intensity ratio of GCaMP to tdTomato >0.15) sustained for at least 30 min. (**e**) Longitudinal analysis of the clinical score of the indicated mouse groups. The data are shown as the mean ± s.e.m. in (**a**) (n = 5), (**d**) (n = 3), and (**e**) (*Spade*: n = 7–12, TRPA1-deficient *Spade*: n = 5–7). *p < 0.05, **p < 0.01.

Having observed epidermal nerve Ca^2+^ increases in the two different models of chronic itch, we next sought to investigate the molecular mechanism of the epidermal nerve Ca^2+^ increases and to examine if blockade of the mechanism could ameliorate the dermatitis itch. Non-selective cation channels of the transient receptor potential (TRP) family could be good candidates for the Ca^2+^ entry pathway in this context. The previous single cell-transcriptome analysis of primary sensory neurons suggested that among the TRP family members, TRPC3 and TRPA1 are the most abundantly expressed in neurons that innervate the SG (Supplementary Fig. 7a)^24^. However, TRPC3 was reported to be dispensable for chemical-induced itch conducted by the SG-innervating neurons^25^. In contrast, TRPA1 was reported to be required for itch of the AEW dry skin model^23^. Therefore, we examined if local inhibition of TRPA1 could attenuate itch in the ear of *Spade* mice. Indeed, topical application of a TRPA1 inhibitor HC-030031 acutely reduced their ear scratch counts (Fig. 7a). Then, we performed intravital Ca^2+^ imaging analysis of epidermal nerves before and after topical application of the inhibitor. We found that sustained Ca^2+^ increases in *Spade* mice were suppressed by the inhibitor (Fig. 7b–d). Finally, in order to investigate the role for TRPA1 in the development of chronic dermatitis of *Spade* mice, we crossed *Spade* mice with TRPA1-deficient mice (Supplementary Fig. 7b,c), and found that scratch lesions of TRPA1-deficient *Spade* mice were significantly ameliorated compared to littermate TRPA1-sufficient *Spade* mice (Fig. 7e). These results indicate that TRPA1 is involved in epidermal nerve Ca^2+^ increases and pathological itch of *Spade* mice.

## Discussion

Our intravital imaging study reveals the dynamic nature of epidermal nerves, demonstrating that they constitutively extend, retract, and get their endings pruned. Previous studies analyzed cutaneous nerve regeneration or remodeling induced by skin injury or hair clipping^26,27^. In addition, the increase of epidermal nerves was reported in the skin of AD patients and mouse models^28–32^, although contradicting observations have also been made^11^. Furthermore, nerve pruning in the body surface tissues has been characterized in Drosophila pupae and Zebrafish during development or regeneration after injury^33–35^. To our knowledge, the present report is the first to characterize constitutive pruning and remodeling of sensory nerves in the adult mammalian epidermis.

Importantly, our results suggest that the epidermal nerve pruning plays a role in the maintenance of their homeostatic positioning under the TJ barrier. It has been proposed that epidermal barrier impairment promotes the exposure of epidermal nerves to external environmental agents to cause AD itch^2,3^. In this model, the cornified layer is important for the protection of epidermal nerves as it covers all the other skin components including nerves. Our study suggests that, in addition to the cornified layer, TJs in the SG are involved in the protection of epidermal nerves from external agents. Consistent with this concept, the itch development in *Spade* mice seemed to coincide with the impairment of their epidermal TJs, while the barrier function of their cornified layer was already weakened at least a couple of weeks before the itch onset^13^.

Our imaging results also suggest that the epidermal nerve pruning takes place as a result of previously unknown interactions between epidermal nerves and keratinocytes. The nerve pruning at intersections with newly forming TJs could be a result of mechanical constriction of nerve fibers by the newly forming TJs. It is also possible that nerves play an active role in the pruning mechanism by inducing molecular changes in their parts to be trimmed in response to the TJ formation around them. The local Ca^2+^ elevations that we observed during pruning might be one of such changes. From our low time-resolution imaging analysis, however, it is not clear whether the Ca^2+^ increases during pruning began before the onset of nerve pruning in the normal epidermis. Therefore, we do not exclude that the Ca^2+^ increases during pruning might be secondary to the plasma membrane disruption in the beginning of pruning. When nerve pruning took place in positions where TJs were not forming, keratinocytes appeared to actively respond to adjacent nerve fibers, because ZO-1-Venus protein transiently accumulated at the site of nerve pruning. In this case as well, whether and how the transient accumulation of ZO-1 and possibly other TJ proteins is mechanistically involved in the nerve severing should be clarified by future researches.

In the *Spade* mouse skin, after the shape of TJ-forming keratinocytes became atypical, the epidermal nerve pruning at newly formed TJs was delayed compared to that in the normal skin, and nerve fibers penetrated TJs without being pruned for hours. Furthermore, repetitive or sustained Ca^2+^ increases in epidermal nerves and Ca^2+^ spikes that reached dermal nerves were induced before and when pruning of the epidermal nerves finally took place. Therefore, it is likely that the nerve fibers showing the aberrant Ca^2+^ increases were penetrating TJs for a prolonged time before pruned. In agreement with this possibility, the frequency of the Ca^2+^ increases preceding pruning in Spade mice in Fig. 5h was consistent with the frequency of nerve fibers that penetrated TJs without getting pruned for prolonged time in Fig. 4h and also with the frequency of TJ-penetrating nerve fibers in the fixed tissue in Fig. 1c. The aberrant Ca^2+^ increases might be caused by environmental stimulations to epidermal nerves that penetrated the TJ barrier. Alternatively, and more intriguingly, it is possible that prolonged interactions between epidermal nerves and compromised TJs might aberrantly activate the nerves to evoke the local Ca^2+^ increases and action potentials.

The SG-innervating neurons were reported to be polymodal nociceptors, which transmit pain and/or itch in response to mechanical, thermal, and chemical stimuli^8,25,36,37^. As *Spade* mice do not show signs of abnormal pain, it is highly possible that the aberrant Ca^2+^ increases in nerve fibers innervating the SG are involved in the induction of *Spade* itch. This possibility is further supported by the observations that both epidermal nerve Ca^2+^ increases and itch were attenuated by the TRPA1 inhibition in *Spade* mice. Future studies need to examine if specific depletion of the SG-innervating neurons or conditional depletion of TRPA1 only in the SG-innervating neurons gives consistent results.

The *Spade* mutation in the Jak1 gene causes hyperactivation of JAK1 in keratinocytes, and the consequent skin barrier impairment appears to be a major cause of the itch development because petrolatum covering of the ear skin delayed the disease onset^13^. In addition, hyperactivation of JAK1 in sensory nerves likely promotes the development and/or exacerbation in the *Spade* dermatitis. Recent studies have found that type-2 cytokines such as IL-4 and IL-31 directly act on sensory neurons that express the cytokine receptors to induce Ca^2+^ increases *ex vivo* and itch in a JAK1-dependent manner^38–42^. Barrier impairment-associated Ca^2+^ increases in epidermal nerves do not necessarily require the Jak1 mutation because they were observed not only in *Spade* mice but also in AEW-treated mice without the Jak1 mutation. Nonetheless, it is plausible that JAK1 hyperactivation in *Spade* sensory nerves contributes to the Ca^2+^ increase and itch, as intensities of both appear to be higher in *Spade* mice than in AEW-treated mice. Thus, it is proposed that in the inflammatory skin milieu, JAK1 activation in both keratinocytes and sensory nerves contributes to sensory nerve activation to transmit itch.

## Methods

### Mice

All experimental protocols were approved by the RIKEN Animal Experiment Committee, and all experiments were performed in accordance with the Regulations and Guidelines for Animal Experiments of RIKEN. *Spade* mice and ZO-1-Venus mice have been previously described^13,19^. C57BL/6 J mice were purchased from CLEA Japan. Nav1.8-Cre mice (EMMA ID: 04582)^14^ were obtained from Medical Research Council. Rosa26-CAG-flox-stop-tdTomato mice (Ai14, Stock No: 007908)^15^, Rosa-CAG-flox-stop-GCaMP3 mice (Ai38, Stock No: 014538)^20^, and Rosa26-flox-stop-DTA mice (ROSA-DTA, Stock No: 009669)^18^ were obtained from the Jackson Laboratory. To generate the TRPA1-deficient mice, the coding region in the first exon of *Trpa1* was replaced with the flippase (Flpo) recombinase gene followed by the SV40 polyA signal and a PGK-Neo cassette flanked by loxP sites (Supplementary Fig. 7b). C57BL/6 J × C57BL/6 N hybrid ES clones that underwent the desired homologous recombination were selected, and injected into BALB/c blastcysts. Chimeric male mice were mated with C57BL/6 J female mice for germline transmission of the targeted *Trpa1* allele. All the mice used for this study were maintained in the specific pathogen-free facility at the RIKEN Yokohama Campus.

### Experimental treatments and behavioral analysis of mice

For preventing *Spade* mice or AEW-treated mice from scratching their ears and/or for preventing painless Nav1.8-DTA mice from injuring their own skin, mice were fitted with plastic Elizabethan collars (Kent Scientific, EC201V-5) after the age of 6 weeks. Collared mice were maintained on sheet type cage bedding PULMAS 3000 (Material Research Center Scitex), because bedding chips would have stuck to their hair that became greasy over time due to their inability to groom. Each collared mouse was euthanized as soon as the behavioral or imaging analysis of the mouse was completed. For the induction of dry skin-associated itch, we performed sebum depletion in the ear skin. For this purpose, we modified the previously described protocol^21^. Mice that had been wearing Elizabethan collars for five days were anesthetized with 5% isoflurane for 20 s and then with 1.5% isoflurane. The ear was gently sandwiched for 30 s between KimWipes (Kimberly Clark) soaked with a 1:1 mixture of acetone and ether, dried up for 30 s, and then sandwiched between water-soaked KimWipes for 30 s. The collared mice received these treatments twice a day (in the morning and evening) for 7 to 9 days until the mice showed significant scratching behavior. To analyze scratching behavior, the behavior of *Spade* mice and AEW-treated mice was recorded on a portable video camera (Panasonic, HC-V360MS-W) for 5 and 30 min, respectively. The number of strokes with the hind paw to scratch the ear was visually counted in the videos. In the experiments to analyze the effect of the TRPA1 inhibitor on Spade itch, the number of scratching strokes by the hind paws was determined by using the MicroAct system (Neuroscience Inc.) in combination with video camera recording. At least a week before the analysis, a Teflon-coated cylindrical magnet (Ø1 mm × 3 mm) was subcutaneously implanted into each of both insteps of isoflurane-anesthetized mice. Small incisions made for the magnet implantation were sealed by Aron Alpha A (Sankyo). Each mouse was placed into a chamber surrounded by a detection coil to measure motions of the implanted magnet for 30 min. Behavior of the mouse was simultaneously recorded by a video camera placed on the transparent lid of the chamber. After the first 30 min recording, 20 μL of ethanol or 20 μL of 620 μM HC-030031 in ethanol was topically applied onto each of both ears (approximately 10 μL onto each side of an ear flap). Then, scratching behavior was recorded for another 30 min as above. Scratching bouts and strokes in each bout were determined by the software of the MicroAct system. The parameters used for the software analysis were as follows: Peak Range 0.3–3 V, P-P Range 0.4–4.5 V, Frequency Range 10–35 Hz, Beats Range 2–1000, and Duration Range 0.105–100 s. By viewing the video footages, ear-scratching bouts were manually extracted from the bouts measured by the MicroAct system for determining the number of ear-scratching strokes.

### Whole mount immunofluorescence staining of the mouse ear skin

Mouse ears were harvested and split into halves. After fixation in 4% paraformaldehyde in PBS for 1 h on ice, the tissues were washed in PBS at 4 °C for 1 h. The subcutaneous cartilage was carefully removed with tweezers under a stereo microscope. The remaining tissues were blocked in the blocking buffer containing 5 mg/ml bovine serum albumin (Wako, 018–15154), 0.3% TritonX-100, 5% fetal bovine serum (SIGMA, 172012-500 ML, Lot#13H469), 2% normal goat serum (Jackson 005-000-001), and 5 μg/ml anti-CD16/CD32 (BD Pharmingen, clone: 2.4G2) in PBS at 37 °C for 18 h, and then incubated with primary antibodies in the blocking buffer at 37 °C for 18 h. As the primary antibodies, mouse anti-ZO-1 (hybridoma clone: T8–754, kindly provided from Dr. Mikio Furuse and Dr. Masahiko Itoh)^43^ was used at a 1:2 dilution of the hybridoma culture supernatant, rabbit anti-PGP9.5 (UltraClone, RA-95101) at a 1:300 dilution, and anti-MBP (Millipore, MAB385) at a 1:100 dilution. After washing with the wash buffer containing 2 mg/ml bovine serum albumin and 0.1% TritonX-100 in PBS at 37 °C for 6 h (2 h × 3 times), the tissues were stained with secondary antibodies in the blocking buffer at 37 °C for 18 h. As the secondary antibodies, goat anti-mouse IgG conjugated with Alexa Flour 488 (Life Technologies, A-11017) was used at a 1:200 dilution, goat anti-rabbit IgG conjugated with Alexa Flour 555 (Life Technologies, A-21428) at a 1:100 dilution, and goat anti-rat IgG conjugated with Alexa Flour 647 (Life Technologies, A-21247) at a 1:100 dilution. After washing with the wash buffer at 37 °C for 4 h, the tissues were stained with 3.5 μM DAPI (Dojindo, D523) in the wash buffer at 37 °C for 1 h, and washed again for 1 h. Specimens were mounted in Fluorescence mounting medium (DAKO, S3023).

### Human sample collection and whole mount staining

The experimental protocols were approved by the Keio University School of Medicine Ethics Committee and the RIKEN Ethics Committee, which stipulate adherence to the Declaration of Helsinki. Prior to sample collection, informed consent was obtained after the nature and possible consequences of the studies were explained to the participants. Healthy skin samples from three 54 to 66-year old Japanese individuals were obtained from tissues excised during surgery of benign skin tumor or from leftover tissues for skin transplantation. AD skin biopsy samples were obtained from three 36 to 62-year old Japanese patients. Non-lesional skin samples of the AD patients were of the skin without visible lesions but adjacent to the lesional skin. Lesional skin samples are of the skin with erythema, induration/lichenification, and excoriation (patient 1), with erythema and edema (patient 2), and with papules (patient 3). After excision, the samples were immediately fixed in 4% paraformaldehyde at 4 °C for 2 h, and washed in PBS at 4 °C for more than 2 h. The fixed samples were cut into thin specimens with roughly 1–2 mm × 1–2 mm of the skin surface and 2–3 mm of the length roughly perpendicular to the surface, and were subjected to staining and tissue clearing using the CUBIC method (Fig. 2B)^44^ or the iDISCO method (Fig. S2D)^45^. To employ the CUBIC method, the specimens were blocked, stained, and washed in the blocking buffer and wash buffer described above in whole mount immunofluorescence staining of the mouse ear skin. The specimens were blocked overnight, incubated with the primary antibodies at 37 °C for 2 days, washed at 37 °C for 6 h (2 h × 3 times), incubated with the secondary antibodies at 37 °C for 2 days, and washed at 37 °C for 4 h. As the primary antibodies, mouse anti-ZO-1 (hybridoma clone: T8–754) was used at a 1:20 dilution of the antibody extract obtained through the Hollow Fiber Bioreactor (FiberCell Systems), and rabbit anti-PGP9.5 (UltraClone, RA95101) at a 1:300 dilution. As the secondary antibodies, goat anti-mouse IgG conjugated with Alexa Fluor 488 (Thermo Fisher Scientific, A-11017) was used at a 1:200 dilution, and goat anti-rabbit IgG conjugated with Alexa Fluor 555 (Thermo Fisher Scientific, A-21428) at a 1:100 dilution. Before tissue clearing, the specimens were stained with 3.5 μM DAPI at 37 °C for a day, and washed at 37 °C for a day. The stained specimens were subjected to tissue clearing as described in the CUBIC protocol^44^. The specimens were immersed in the Reagent 1 solution at 37 °C for 2 days. After washing with PBS at 37 °C for 6 h (2 h × 3 times), the tissues were immersed in the Reagent 2 solution at 37 °C for 2 days. The specimens were stored and observed in the Reagent 2 solution at room temperature. To employ the iDISCO method, methanol bleaching, and whole-mount immunofluorescence staining were conducted as previously described^45^. The blocking, staining, and wash buffer solutions were prepared as described in the iDISCO protocol except that the blocking and staining buffer solutions contained 3–6% normal goat serum instead of normal donkey serum. The specimens were blocked overnight, incubated with the primary antibodies at 37 °C for 2 days, washed at 37 °C for a day, incubated with the secondary antibodies at 37 °C for 2 days, and washed at 37 °C for 5 h. As the primary antibodies, mouse anti-ZO-1 (hybridoma clone: T8–754) was used at a 1:20 dilution of the antibody extract obtained through the Hollow Fiber Bioreactor (FiberCell Systems), and rabbit anti-PGP9.5 (Enzo Life Science, ADI9055201) at a 1:100 dilution. As the secondary antibodies, goat anti-mouse IgG conjugated with Alexa Fluor 633 (Thermo Fisher Scientific, A-21052) and goat anti-rabbit IgG conjugated with Alexa Fluor 555 (Thermo Fisher Scientific, A-21428) were used at a 1:100 dilution. Before going to the tissue clearing step, the specimens were stained with 3.5 μM DAPI in the staining buffer solution at 37 °C for a day, and washed with the wash buffer solution at 37 °C for a day. Tissue clearing was performed as described in the iDISCO protocol^45^.

### Confocal microscopy of whole-mount tissue samples

Whole-mount tissue images were obtained using the TCS SP5 II or TCS SP8 confocal microscope equipped with HC PL APO 20×/0.75 CS2 and HC PL APO 63×/1.40 OIL CS2 objective lenses (Leica Microsystems). Specimens were scanned sequentially with a 405-nm diode laser, a 488-nm argon laser, a 543-nm He-Ne laser or a 561-nm diode laser, and a 633-nm He-Ne laser. Emission signals were collected by using a 440–480-nm emission filter for DAPI, a 504–538-nm emission filter for Alexa Flour 488, a 567–607-nm emission filter for Alexa Flour 555 or tdTomato, and a 658–780-nm emission filter for Alexa Flour 633 or 647. For image acquisition, 61.5–582 μm × 61.5–582 μm *x–y* planes were scanned at a resolution of 0.06–0.57 μm per pixel, and image stacks of 37–201 *x–y* planes with 0.5-μm *z* spacing were constructed after averaging 3–4 frames for each *x–y* plane.

### Intravital multiphoton microscopy

For visualization of Keratinocyte nuclei, we injected 10 μl of 100 μg/ml Hoechst33342 (Thermo Fisher Scientific, H3570, diluted in saline) intradermally in the ear skin at least 2 h before imaging. Intravital imaging of ear pinnae was performed as described in the previous study^46^ with some modifications. Mice were anesthetized with isoflurane (Wako; 5% for induction, 1.5% for maintenance) in a stream of oxygen, and placed on an imaging platform (LEICA MATS, Tokai Hit) adjusted to 37 °C. The dorsal or ventral side of the ear pinna wetted with water was gently immobilized by vinyl tape on a coverslip which was glued with High Silicone II (Denken-Highdental) over the hole of the imaging platform. Imaging was conducted for the area where hair follicles were sparse. Images were acquired using an inverted TCS SP8 multiphoton microscope (Leica Microsystems) as described previously^47^, with modifications as follows: a 460/50-nm emission filter was used for Hoechst33342 and second harmonic generation of collagen fibers; a 525/50-nm emission filter was used for GCaMP3; for image acquisition, 93.2–155 μm × 93.2–155 μm x–y planes were scanned at a resolution of 0.18–0.3-μm per pixel and images of 18–85 *x–y* planes with 1.0-μm *z* spacing were constructed after averaging 2–3 video frames for each *x–y* plane; 3D stacks were acquired every 90 s. Data sets among which fluorescence intensities needed to be comparatively analyzed were acquired using identical settings of the laser power and detector voltage. For analyzing the effect of the TRPA1 inhibitor on nerve Ca^2+^ increases, the dorsal side of the ear was analyzed by imaging. Two image stacks of Nav1.8-tdTomato/GCaMP3 signals in the SG were obtained, with a 30-min interval. Then, the mouse was detached from the scope stage, and 10 μL of 70% ethanol was topically applied onto the dorsal side of the ear. The mouse was replaced back on the scope stage, and two image stacks with a 30-min interval were again obtained at a skin position as close as possible to the original position in 15 min after 70% ethanol application. The same process was repeated with 10 μL of 620 μM HC-030031 in 70% ethanol.

### Intravital confocal microscopy

For intravital imaging of ZO-1-Venus and nerve fibers, we used the TCS SP8 confocal microscope with the HC PL IRAPO 40×/1.10 W CORR CS2 objective lens, because the z-axis resolution of multiphoton microscopy was not high enough to characterize *in vivo* ZO-1-Venus localization in the SG, which was close enough from the skin surface to be visualized by single-photon confocal microscopy. Venus and tdTomato signals were obtained through 504–538 and 567–607 nm emission filters by using 488-nm argon and 561-nm diode lasers, respectively. For image acquisition, 145 μm × 145 μm x–y planes were scanned at a resolution of 0.14-μm per pixel and images of 37–41 *x–y* planes with 0.5-μm *z* spacing were constructed after averaging 4 video frames for each *x–y* plane. 3D stacks were acquired every 360–600 s.

### Image analysis

Whole-mount images and intravital images were volume-rendered and analyzed using Imaris 8.1.2 or 8.4.1 (Bitplane). Tracking of the extension and retraction of epidermal nerve endings was performed manually by using the “Spots” function of Imaris MeasurementPro. To obtain fluorescence intensity ratios of GCaMP3 to tdTomato in nerve endings, cubic regions (2^3^ μm^3^) were made in 3D images of Red channel fluorescence signals alone without watching Green channel fluorescence signals to eliminate a bias in making regions, and then mean fluorescence intensities in both channels in each cubic region were obtained.

### RT-qPCR

Four cervical DRGs (C1, C2) from each mouse were harvested and dunked in 1 mL Trizol (Thermo Fisher Scientific) in a screw cap tube (WATSON,1392-200-c) containing a Zirconia Bead (TOMY, ZB-50). The DRGs were crushed by MicroSmash (TOMY, MS-100R) at 4,000 rpm at 4 °C for 1 min. The lysate was transferred to the fresh tube and added with 200 mL chloroform. The tubes were vigorously shaken by hand for 15 seconds, incubated at room temperature for 3 min, and then centrifuged at 12,000 × g at 4 °C for 15 min. The aqueous phase was transferred to a fresh tube and added with 1 mL 20 mg/mL RNA free Glycogen Solution (GMbiolab, GM14) and 500 mL isopropyl alcohol. After gently inverted five times and incubated for 10 min at room temperature, the samples were centrifuged at 12,000 × g at 4 °C for 10 min, and then the supernatant was discarded. The pellets were washed with RNase-free 70% ethanol, dried up for 10 min, and dissolved in 50 mL DEPC-treated water (Nacalai tesque, 36415-54). The RNA concentration was measured by using Fusion α using Quant-iT RiboGreen RNA Assay Kit (Thermo Fisher Scientific, R11490). The RNA quality was analyzed by using Agilent RNA 6000 pico Kit (Agilent Technologies, 5067-1513) and Agilent 2100 bioanalyzer (Agilent Technologies). cDNA was synthesized from 50 ng RNA using SuperScript III First-Strand Synthesis System (Thermo Fisher Scientific, 18080051). Real-time PCR analysis was performed by using Fast SYBR Green Master Mix (Thermo Fisher Scientific, 438561) and Step One Plus Real-Time PCR System (Applied Biosystems) with the following condition: 95 °C for 20 seconds followed by 40 cycles of 95 °C for 3 seconds and 60 °C for 30 seconds. The primers used for amplification of the *Trpa1* and *Gapdh* cDNA fragments were as follows.

Trpa1-Ex14 F1: ACAGCACTCCACTTTGCAGC

Trpa1-Ex15 R1: ATCTCCATGATTGGACATCG

Trpa1-Ex22 F1: CATTGCTGAGATCGACCGGAG

Trpa1-Ex23 R1: TGTGAAGGCAATAAGCTGCC

Gapdh-F: ATGGTGAAGGTCGGTGTGAACGGATTTGGC

Gapdh-R: AGCTTCCCATTCTCGGCCTGGACTGTTCTG

### Statistics

Probabilities were determined by two-tailed Student’s t test for the comparison of two means and by one-way ANOVA with Bonferroni’s post-test for the comparison of more than two means, calculated by Prism 7 (GraphPad Software).

## Supplementary information

Supplementary Information

Supplementary Movie 1

Supplementary Movie 2

Supplementary Movie 3

Supplementary Movie 4

Supplementary Movie 5

Supplementary Movie 6

Supplementary Movie 7

Supplementary Movie 8

Supplementary Movie 9

Supplementary Movie 10

Supplementary Movie 11

Supplementary Movie 12

Supplementary Movie 13

Supplementary Movie 14

Supplementary Movie 15
