# Supplementary material for: Homeostatic pruning and activity of epidermal nerves are dysregulated in barrier-impaired skin during chronic itch development
Source: Sci Rep. 2019 Jun 13;9:8625. doi: 10.1038/s41598-019-44866-0 (PMC6565750; doi:10.1038/s41598-019-44866-0)
Supplement: Supplementary file 1 — Supplementary Information [file 41598_2019_44866_MOESM1_ESM.pdf]

Supplementary Information

Homeostatic pruning and activity of epidermal nerves are dysregulated in barrier-impaired skin during chronic itch development

Authors

Sonoko Takahashi, Azusa Ishida, Akiharu Kubo, Hiroshi Kawasaki, Sotaro Ochiai, Manabu Nakayama, Haruhiko Koseki, Masayuki Amagai, and Takaharu Okada*

*Correspondence to: takaharu.okada@ riken.jp

Contents

・Supplementary Figures 1-7 and Legends

・Supplementary Movie Legends

Supplementary Figures and Legends

Supplementary Figure 1

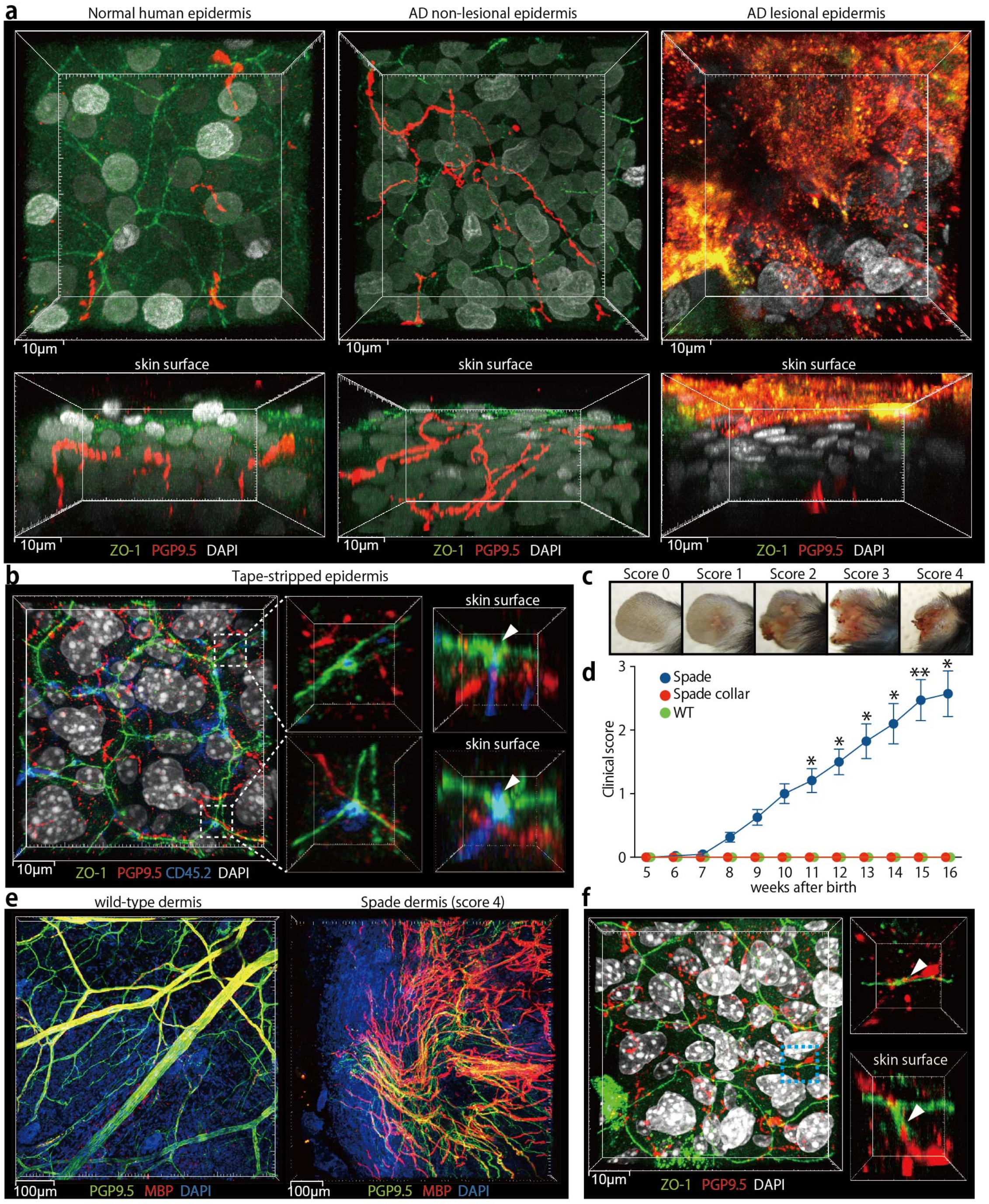

Supplementary Fig. 1: Supplementary data for Figure 1

(a) Additional vertical and horizontal projection images of epidermal nerves and TJs in the normal human skin and AD skin. **(b)** Whole-mount confocal fluorescence images of the epidermis of the WT mouse skin subjected to tape stripping. The skin sample was collected after 12h of tape stripping. The rectangles in the large field vertical projection image (far-left, 9.5μm projection depth) indicate regions in which Langerhans cell processes penetrate the TJs, as shown in the magnified vertical projection images (middle, 8.5μm projection depth) and horizontal projection images (far-right, 9μm projection depth). **(c)** Representative images for lesion severity scoring of *Spade* dermatitis in each ear, i.e., score 0: no lesion, score 1: small lesion in the middle of the earflap, score 2: large lesion spreading to the earflap edge, score 3: severe lesion accompanied with the loss of a part of the earflap, score 4: loss of a large part of the earflap. The score of each mouse was determined as the average of lesion severity scores of two ears. **(d)** Longitudinal analysis of the clinical score of the indicated mouse groups. Elizabethan collars were used to prevent the indicated mouse group from scratching the ears after 7 weeks of age. The data are shown as the mean ± s.e.m. (n = 8–37). *$p < 0.05$, **$p < 0.01$. **(e)** Whole-mount confocal fluorescence images of the ear dermis from a WT mouse and a *Spade* mouse with severe lesions (lesion severity score 3). **(f)** Whole-mount confocal fluorescence images of the epidermis from a *Spade* mouse that had worn an Elizabethan collar. Magnified vertical (upper right) and horizontal (lower right) views of the blue dashed square region are also shown. Filled arrowheads indicate ZO-1 accumulation around the nerve fiber.

Supplementary Figure 2

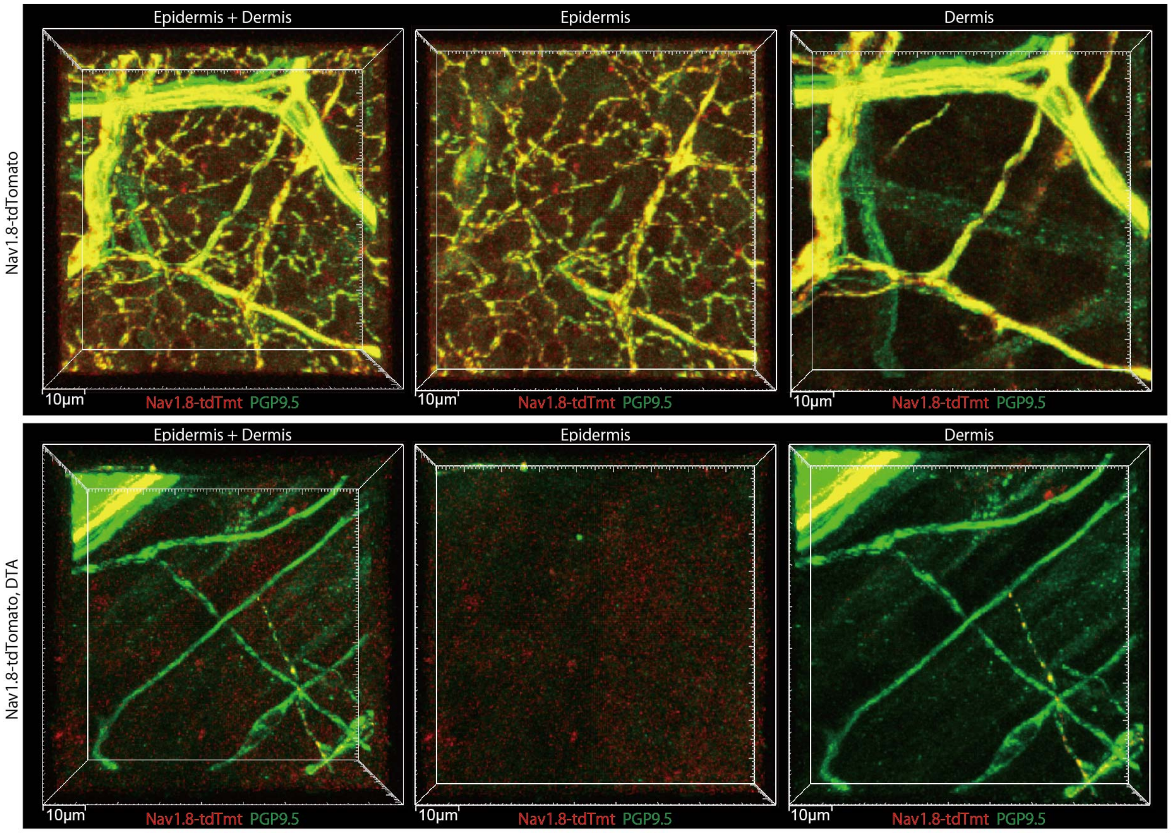

Supplementary Fig. 2: Supplementary images for Figure 2.

Magnified images of the epidermal and dermal nerves shown in Fig. 2a. Note that all the epidermal nerves are Nav1.8-tdTomato^{+}.

Supplementary Figure 3

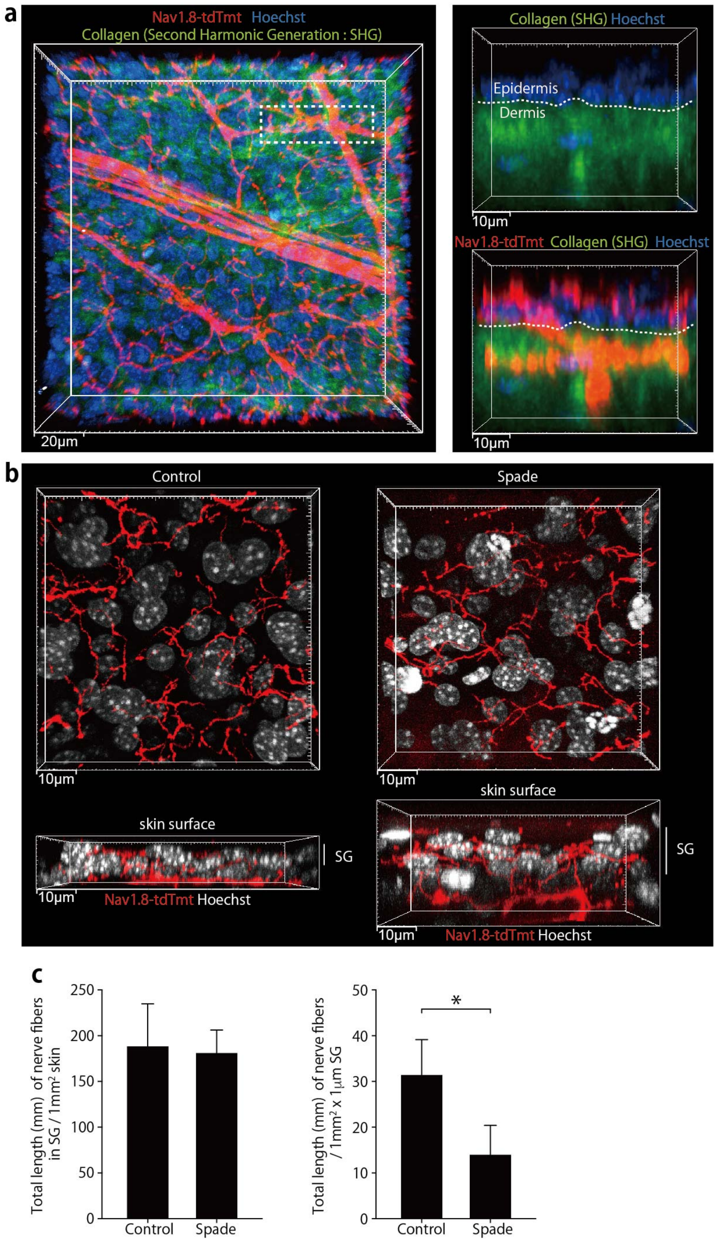

Supplementary Fig. 3: Supplementary data for Figure 3.

Intravital multiphoton images of the ear skin of an Nav1.8-tdTomato mouse. **(a)** Vertical projection view (left panel, 49μm projection depth) and horizontal projection views (right panel) of the region demarcated by the dashed rectangle in the left panel. **(b)** Intravital confocal images of the ear skin of control Nav1.8-tdTomato and *Spade* Nav1.8-tdTomato

mice. The upper panels show vertical projection views of the SG (6 μm projection depth for the control mouse, 15 μm projection depth for the Spade mouse). The lower panels show horizontal projection views of the epidermis and dermis (20 μm projection depth for both). **(c)** The left histogram shows the total length of nerve fibers in the SG (6 μm thick in the control skin, 14 ± 3 μm thick in the Spade skin). The right histogram shows the nerve fiber length normalized by the SG thickness. The data are shown as the mean ± s.e.m. (n = 3). $*p < 0.05$.

Supplementary Figure 4

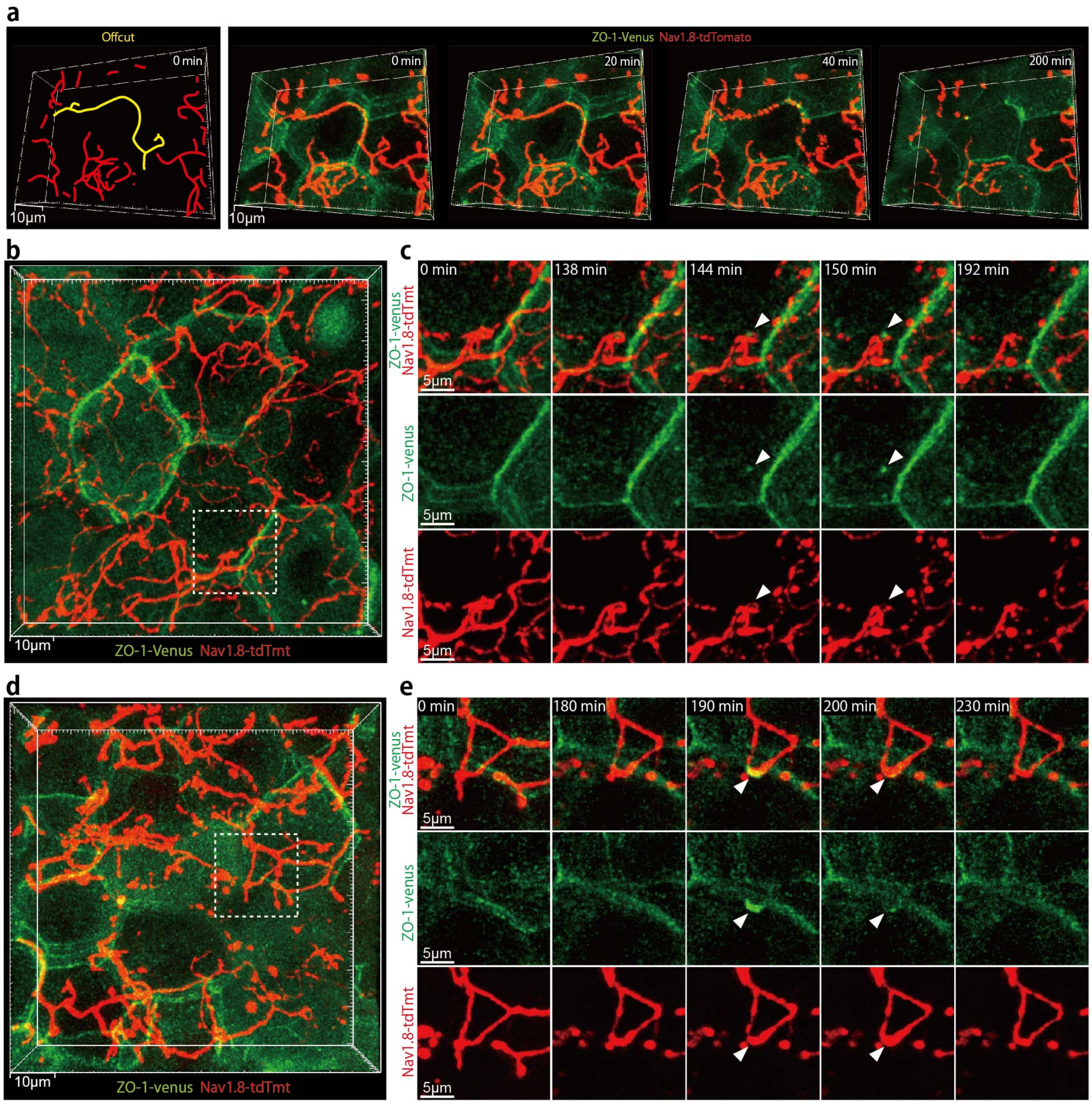

Supplementary Fig. 4: Supplementary images for Figure 4.

(a) In vivo time-lapse confocal images of an epidermal nerve offcut in the ear skin of a *Spade* Nav1.8-tdTomato ZO-1-venus mouse (three right panels). See also Supplementary Movie 9. In the far-left drawings, the nerve offcut is indicated by the yellow lines. **(b)** An intravital confocal image (9.5 μm projection depth) of epidermal nerves and TJs in an Nav1.8-tdTomato ZO-1-Venus mouse. **(c)** Time-lapse images (8.5 μm projection depth) of

transient, punctate ZO-1-Venus accumulation (arrowheads) at the site of nerve pruning in the region demarcated by the dashed square in **b**. See also Supplementary Movie 10. **(d)** An intravital confocal image (20 μm projection depth) of epidermal nerves and TJs in a *Spade* Nav1.8-tdTomato ZO-1-Venus mouse. **(e)** Time-lapse images (8.5 μm projection depth) of transient, punctate ZO-1-Venus accumulation (arrowheads) on a nerve branch that did not undergo pruning in the region demarcated by the dashed square in **d**. See also Supplementary Movie 11.

Supplementary Figure 5

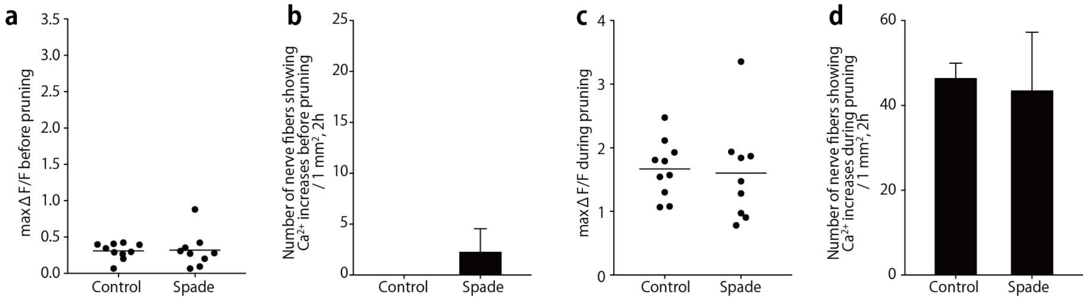

Supplementary Fig. 5: Supplementary data for Figure 5.

(a) Maximum fluorescence intensity changes in the GCaMP3 channel before pruning in young Spade mice at 4-6 weeks old. **(b)** The area- and time-normalized number of nerve fibers showing Ca^{2+} increases (max $\Delta F/F > 0.5$) before pruning. **(c)** Maximum fluorescence intensity changes in the GCaMP3 channel during pruning. **(d)** The area- and time-normalized number of nerve fibers showing Ca^{2+} increases (max $\Delta F/F > 0.5$) during pruning. The data are shown as the mean ± s.e.m. (n = 3).

Supplementary Figure 6

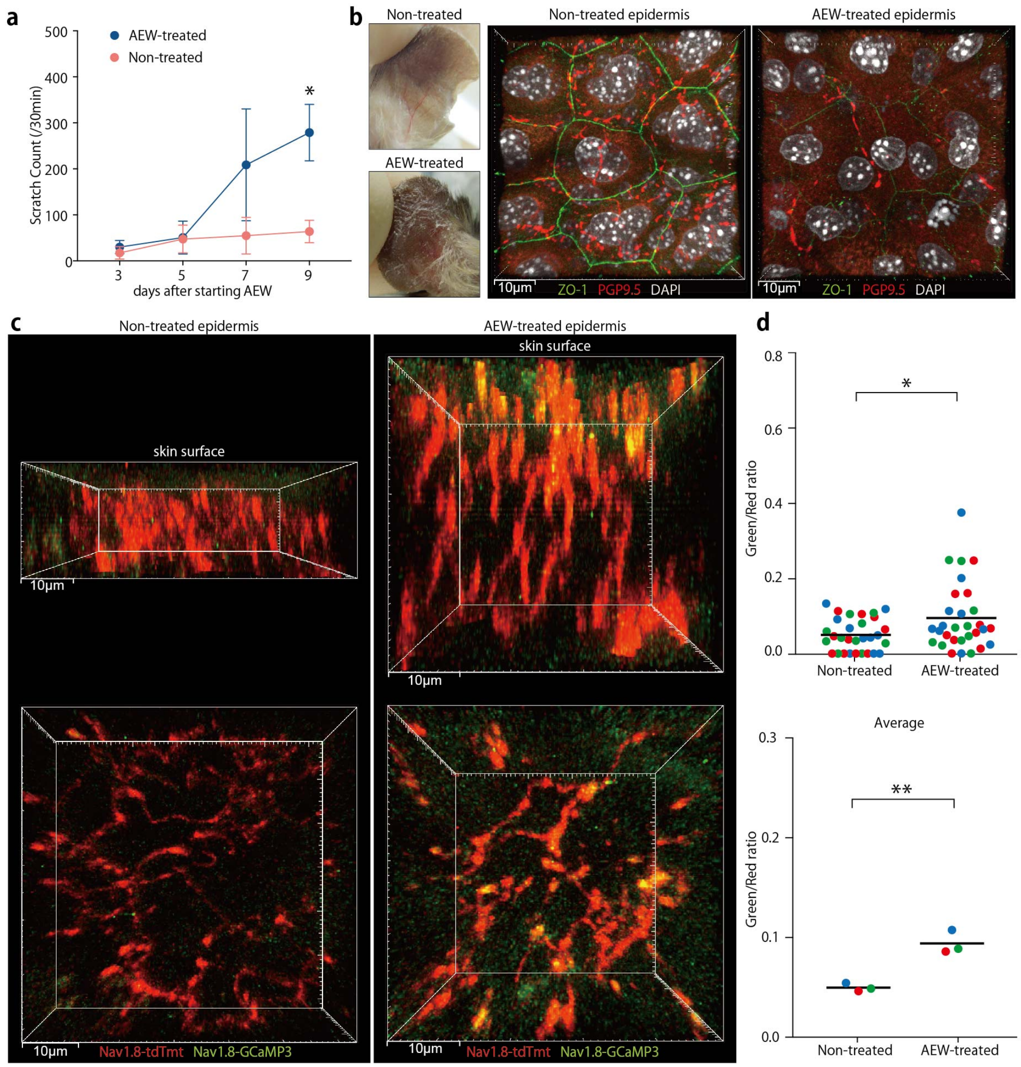

Supplementary Fig. 6: Supplementary data for Figure 6.

(a) The number of scratching strokes by the hind paws of Elizabethan collar-wearing mice with or without the AEW treatment. The data are shown as the mean ± s.e.m. (n = 3). **(b)** Images of the ear skin and whole-mount confocal fluorescence images of the ear epidermis with or without the 9-day AEW treatment. **(c)** Intravital multiphoton images of the ear epidermis with or without the 9-day AEW treatment. Upper and lower images are horizontal and vertical projection views, respectively. **(d)** Fluorescence intensity ratio of GCaMP3 to tdTomato in epidermal nerves. $*p < 0.05$, $**p < 0.01$.

Supplementary Figure 7

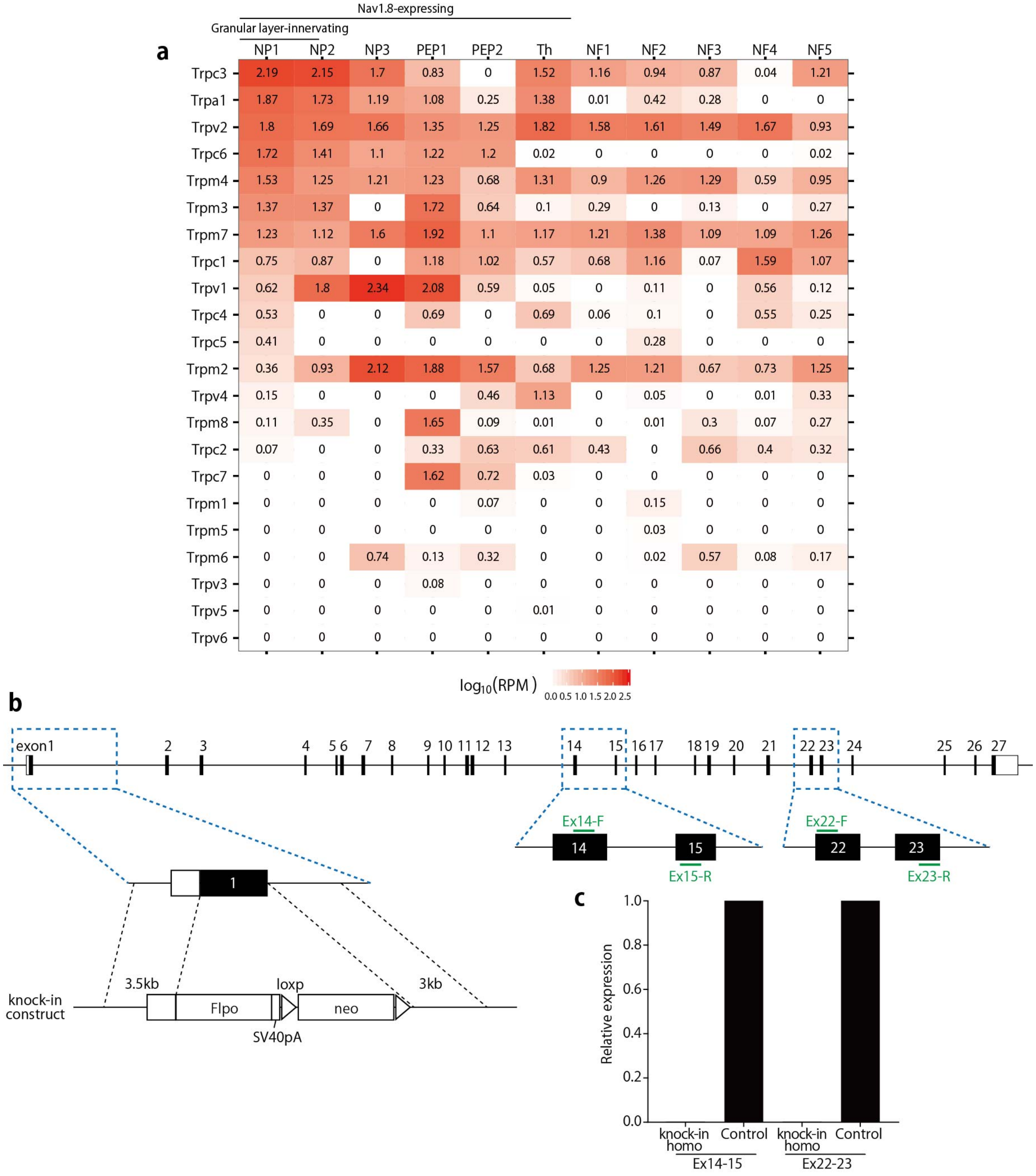

Supplementary Fig. 7: Supplementary data and diagrams for Figure 7.

(a) TRP family gene expression in subpopulations of sensory neurons based on the published single-cell RNA-sequence data from Usoskin et al. 2015 (doi:10.1038/nn.3881). The heat map and the numbers in it show the common logarithm of Reads Per Million

Reads (RPM) of each gene. **(b)** Schematic diagrams of the Trpa1 locus and targeting construct to replace the exon 1 with the flippase (Flpo) recombinase gene and the neomycin resistance gene (neo) flanked by loxp sites. Positions of the primers used for RT-qPCR are also shown. The exons 22 and 23 encode the pore-forming region. **(c)** Relative expression of *Trpa1* mRNA in cervical dorsal root ganglia, which contain sensory neurons innervating the ear skin, of WT mice and mice homozygous for the knock-in Trpa1 allele, as determined by RT-qPCR.

Supplementary Movie Legends

Supplementary Movie 1: 3D-reconstructed images of human epidermal TJs, nerve fibers, and cell nuclei in the normal skin and AD skin.

Supplementary Movie 2: 3D-reconstructed images of epidermal TJs, nerve fibers, and cell nuclei in the WT mouse ear skin and *Spade* mouse ear skin.

Supplementary Movie 3: Dynamic sensory nerve endings in the SG of the normal epidermis. Z-projection depth: 46 μm. Elapsed time is shown as h:mm:ss.

Supplementary Movie 4: Epidermal nerve pruning in the normal mouse skin. Z-projection depth: 14 μm. Elapsed time is shown as h:mm:ss.

Supplementary Movie 5: Tight junction turnover and epidermal nerve dynamics in the control mouse skin. Z-projection depth: 18 μm. Elapsed time is shown as h:mm:ss.

Supplementary Movie 6: Epidermal nerve pruning at intersections with a newly forming tight junction. Z-projection depth: 13.5 μm. Elapsed time is shown as h:mm:ss.

Supplementary Movie 7: Tight junction turnover and epidermal nerve dynamics in the *Spade* mouse skin (score 1). Z-projection depth: 20 μm. Elapsed time is shown as h:mm:ss.

Supplementary Movie 8: A nerve fiber penetrating a TJ without being pruned in the *Spade* mouse skin (score 1). Z-projection depth: 18.5 μm. Elapsed time is shown as h:mm:ss.

Supplementary Movie 9: Degradation of a nerve fiber offcut in the *Spade* mouse skin. Z-projection depth: 16.4 μm. Elapsed time is shown as h:mm:ss.

Supplementary Movie 10: Nerve fiber pruning at a transient, punctate ZO-1 accumulation in the control mouse skin. Z-projection depth: 13 μm. Elapsed time is shown as h:mm:ss.

Supplementary Movie 11: Transient, punctate ZO-1 accumulation on a nerve fiber without pruning the nerve fiber in the *Spade* mouse skin. Z-projection depth: 20 μm. Elapsed time is shown as h:mm:ss.

Supplementary Movie 12: Local Ca^{2+} increases in pruned nerve endings in the normal mouse skin. The lower panels show horizontal views of the region demarcated by the dashed rectangles in the upper panels. Z-projection depth: 19 μm. Elapsed time is shown as h:mm:ss.

Supplementary Movie 13: Repetitive Ca^{2+} increases in an epidermal nerve branch that underwent pruning and a Ca^{2+} spike that reached a dermal nerve fiber in the *Spade* mouse skin (score 0). The lower panels show horizontal views of the region demarcated by the dashed rectangles in the upper panels. Z-projection depth: 47 μm. Elapsed time is shown as h:mm:ss.

Supplementary Movie 14: Ca^{2+} increases in epidermal nerves of the *Spade* mouse ear skin (score 0). Upper images indicate the horizontal view of each bottom images. Z-projection depth: 11 μm (control) and 75 μm (Spade). Elapsed time is shown as h:mm:ss.

Supplementary Movie 15: Ca^{2+} oscillation in an epidermal nerve ending (left) and fragmented epidermal nerve fibers (right) in the Spade mouse ear skin (score 0). Z-projection depth: 45 μm. Elapsed time is shown as h:mm:ss.
